# Cellular mechanism of action of 2-nitroimidazoles as hypoxia-selective therapeutic agents

**DOI:** 10.1016/j.redox.2022.102300

**Published:** 2022-03-21

**Authors:** Faisal Bin Rashed, Diana Diaz-Dussan, Fatemeh Mashayekhi, Dawn Macdonald, Patrick Nicholas Nation, Xiao-Hong Yang, Sargun Sokhi, Alexandru Cezar Stoica, Hassan El-Saidi, Carolynne Ricardo, Ravin Narain, Ismail Hassan Ismail, Leonard Irving Wiebe, Piyush Kumar, Michael Weinfeld

**Affiliations:** a Department of Oncology, University of Alberta, Edmonton, Alberta, AB T6G 2R3, Canada; b Department of Chemical & Materials Engineering, University of Alberta, Edmonton, Alberta, AB T6G 2R3, Canada; c Department of Laboratory Medicine & Pathology, University of Alberta, Edmonton, Alberta, AB T6G 2R3, Canada; d Department of Pharmaceutical Chemistry, Faculty of Pharmacy, University of Alexandria, El Sultan Hussein St. Azarita, Alexandria, Egypt; e Biophysics Department, Faculty of Science, Cairo University, Cairo, Egypt

**Keywords:** Hypoxia, Nitroimidazole, Head and neck tumour, Replication stress, BrdU, 5-Bromo-2’-deoxyuridine, CldU, 5-Chloro-2’-deoxyuridine, CPT, (S)-(+)-Camptothecin, CVS, Crystal violet staining, DTNB, 5,5´-Dithio-bis-2-(nitrobenzoic acid), EdU, 5-Ethynyl-2´-deoxyuridine, FAZA, Fluoroazomycin arabinofuranoside, GAPDH, Glyceraldehyde-3-phosphate dehydrogenase, GST, Glutathione S-transferase, HAP, Hypoxia-activated prodrug, HNC, Head & neck cancer, HU, Hydroxyurea, IAZA, Iodoazomycin arabinofuranoside, IdU, 5-Iodo-2′-deoxyuridine, MTT, 3-(4,5-Dimethylthiazol-2-yl)-2,5-diphenyltetrazolium bromide, N_3_-AZA, Azidoazomycin arabinofuranoside, NAC, N-Acetyl-L-cysteine, NI, Nitroimidazole, PCNA, Proliferating cell nuclear antigen, PI, Propidum iodide, RPA, Replication protein A, TARDCS, Trapped in agarose DNA click staining, TARDIS, Trapped in agarose DNA immunostaining, TNB, 5-Thio-2-nitrobenzoic acid, γ-H2AX, Phosphorylated histone variant H2A.X (Ser139)

## Abstract

Solid tumours are often poorly oxygenated, which confers resistance to standard treatment modalities. Targeting hypoxic tumours requires compounds, such as nitroimidazoles (NIs), equipped with the ability to reach and become activated within diffusion limited tumour niches. NIs become selectively entrapped in hypoxic cells through bioreductive activation, and have shown promise as hypoxia directed therapeutics. However, little is known about their mechanism of action, hindering the broader clinical usage of NIs. Iodoazomycin arabinofuranoside (IAZA) and fluoroazomycin arabinofuranoside (FAZA) are clinically validated 2-NI hypoxic radiotracers with excellent tumour uptake properties. Hypoxic cancer cells have also shown preferential susceptibility to IAZA and FAZA treatment, making them ideal candidates for an in-depth study in a therapeutic setting. Using a head and neck cancer model, we show that hypoxic cells display higher sensitivity to IAZA and FAZA, where the drugs alter cell morphology, compromise DNA replication, slow down cell cycle progression and induce replication stress, ultimately leading to cytostasis. Effects of IAZA and FAZA on target cellular macromolecules (DNA, proteins and glutathione) were characterized to uncover potential mechanism(s) of action. Covalent binding of these NIs was only observed to cellular proteins, but not to DNA, under hypoxia. While protein levels remained unaffected, catalytic activities of NI target proteins, such as the glycolytic enzyme glyceraldehyde-3-phosphate dehydrogenase (GAPDH) and the detoxification enzyme glutathione S-transferase (GST) were significantly curtailed in response to drug treatment under hypoxia. Intraperitoneal administration of IAZA was well-tolerated in mice and produced early (but transient) growth inhibition of subcutaneous mouse tumours.

## Highlights

• Hypoxic cells display preferential sensitivity to IAZA and FAZA.
• They alter cell morphology and induce cytostasis.
• IAZA and FAZA generate covalent adducts of proteins but not DNA.
• GAPDH and GST activities, but not protein levels, are significantly reduced.

## 1 Introduction

Hypoxia or low oxygen tension is a characteristic feature of solid malignancies. It arises from an imbalance between O_2_ consumption and supply, which is triggered by rapid proliferation of cancer cells and aberrant tumour vasculature [1,2]. Hypoxia-induced cellular adaptations confer resistance to radiation, chemotherapy and immunotherapy, leading to poor local control and overall survival [3,4]. This underscores the importance of developing hypoxia-directed therapeutics for effective management of solid tumours.

Nitroimidazoles (NIs) are a class of electron-affinic molecules that are taken up by cells via diffusion and chemically reduced by various cellular nitroreductases, generating nitroradical anions. When cells are well-oxygenated, the nitroradical anions react with intra-cellular O_2_ and revert to the parent molecule, which thereafter freely diffuses out of the cell. In the absence of O_2_ (i.e. under hypoxia), the reduced NIs undergo further reduction to generate reactive hydroxylamine that binds to cellular macromolecules (such as DNA, proteins and glutathione), thereby trapping the drug inside hypoxic cells [5,6]. Due to their preferential accumulation in O_2_ starved tissue, NIs have long been pursued as potential hypoxia-directed therapeutics. However, clinical trials with NIs have not been particularly successful. With the exception of nimorazole [7], most NIs failed to provide therapeutic benefit in a clinical setting [8,9]. Regardless, retrospective analysis of these studies found an improved outcome in patients with hypoxic tumours [10,11]. This supports the notion that NIs can improve therapy outcome if patients are pre-selected based on their tumour oxygenation status. However, our poor understanding of the precise molecular mechanism of NIs makes it challenging to study them in a clinical setting.

**Fig. 1:**
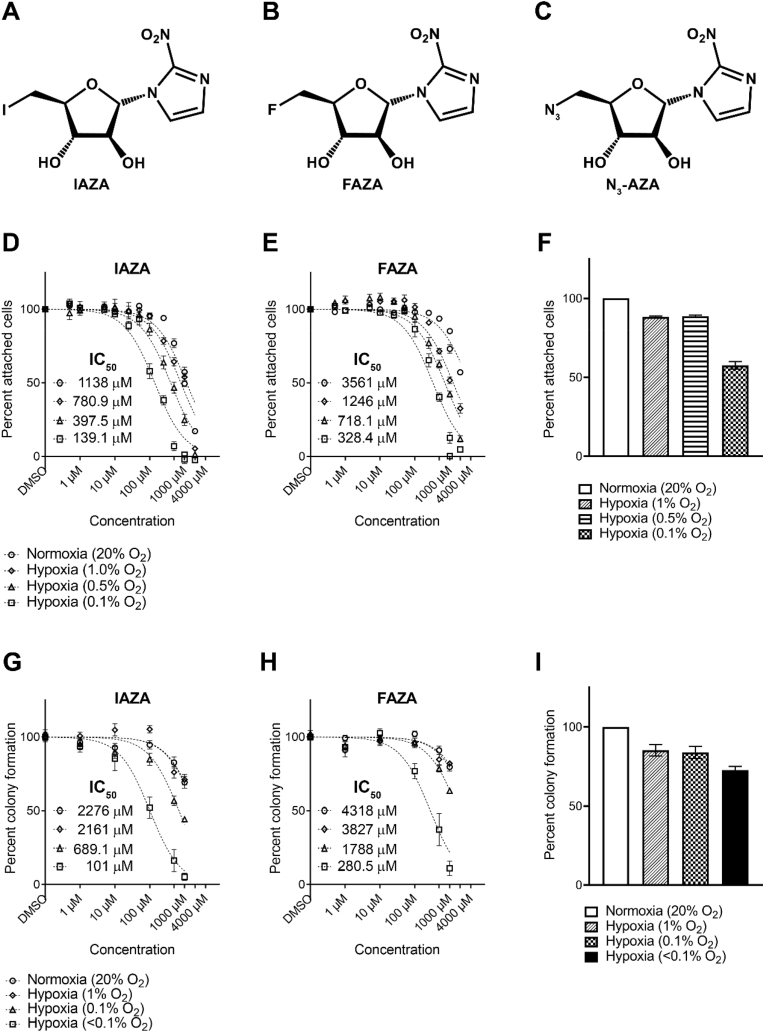
**Hypoxic cells show preferential sensitivity towards IAZA and FAZA treatment.** Chemical formula for IAZA (**A**) FAZA (**B**) and N_3_-AZA (**C**). Crystal violet staining assays were performed with FaDu cells under different O_2_ levels (20%, 1%, 0.5% or 0.1% O_2_). Cells were more sensitive to drug treatment under low O_2_ levels (**D** and **E**). Colony formation assays with FaDu cells measured clonogenicity in response to drug treatment under normoxia (20% O_2_) or different levels of hypoxia (1%, 0.1% or <0.1% O_2_). Cells showed the most sensitivity to treatment when cultured under <0.1% O_2_ (**G** and **H**). IAZA was more toxic than FAZA at the same concentrations. Effects of different levels of O_2_ on cell viability (**F)** and clonogenicity (**I**) are shown. Data represent mean ± S.E.M. from at least three independent experiments.

Iodoazomycin arabinofuranoside (IAZA) and fluoroazomycin arabinofuranoside (FAZA) are 2-NI based compounds that share a common backbone: an azomycin moiety for hypoxia targeting, a pentose sugar to facilitate cell uptake, and a halogen moiety suitable for radiolabelling to impart PET or SPECT imaging (and potential molecular radiotherapy for IAZA) capacity (Fig. 1A-**B**). Originally developed as hypoxic radiotracers, radiolabeled IAZA and FAZA have been chemically characterized in the literature [12,13], and clinically validated as non-invasive hypoxia diagnostics [[14], [15], [16]]. Interestingly, both compounds displayed promising anti-tumour properties under hypoxia in early cell-based assays [12,17,18], necessitating a more in-depth study of their therapeutic potentials. Therefore, using IAZA and FAZA as representative compounds, this study aims at characterizing the anti-tumour effects of 2-NIs, with a particular emphasis on identifying the cellular phenotype induced by these drugs as well as analyzing their effects on target cellular macromolecules.

## 2 Materials and methods

### 2.1 Cell culture

All cell lines (FaDu, A549, PC3 and HCT116) were purchased directly from the American Type Culture Collection (Manassas, VA), and routinely tested for mycoplasma contamination. Cells were grown in DMEM/F-12 medium supplemented with 10% fetal bovine serum, 1% 2 mM l-glutamine and 1% penicillin streptomycin. Cells in culture were maintained for a maximum of 2 months (∼25–30 passages) in a humidified incubator at 37 °C with 5% CO_2_. A humidified chamber equipped with controlled O_2_ flow was used for hypoxia levels up to 0.1% O_2_ (ProOx P110, BioSpherix, Parish, NY). An in-house degassing/regassing system, fitted with metal canisters, was used to achieve O_2_ levels below 0.1% [19]. Glass petri dishes were used to carry out hypoxic experiments in the canisters.

### 2.2 Drug stock preparation

Synthesis of IAZA [12], FAZA [13] and azidoazomycin arabinofuranoside (N_3_-AZA) [20] have been described previously. IAZA, FAZA, N_3_-AZA and Ferrostatin-1 (SML0583, Sigma-Aldrich, Oakville, ON) were prepared as 500 mM solutions in DMSO and stored at −20 °C. *N*-Acetyl-l-cysteine (NAC; A7250, Sigma-Aldrich) was dissolved in 1:1 ratio of DMSO and distilled H_2_O to prepare a fresh 250 mM stock solution for each experiment and added to medium to a final concentration of 3 mM; medium was buffered to ∼pH 7.4 before adding to cells. Stock solutions of 1 M hydroxyurea (HU; H8627, Sigma-Aldrich) and 10 mM (S)-(+)-Camptothecin (CPT; C9911, Sigma-Aldrich) were prepared in DMSO and kept at −20 °C.

### 2.3 Crystal violet staining (CVS) assay

Cells seeded in 96-well plates (Cellstar®; 655180, Sigma-Aldrich) were treated with increasing concentrations of IAZA or FAZA (up to 2000 μM) for 72 h under normoxia (20% O_2_) or hypoxia (1%, 0.5% and 0.1% O_2_); 0.02% DMSO was used as vehicle control. Afterwards, medium was removed, and attached cells were stained with 0.05% crystal violet (C6158, Sigma-Aldrich), washed and air dried for 24 h. Modifications to this standard protocol include: (a) treatment with 100 μM Ferrostatin-1 in addition to IAZA and FAZA for the ferroptosis inhibition assay, and (b) pre-incubation with 3 mM NAC for 2 h for reactive oxygen species (ROS) scavenger assay, after which medium was removed and treatment with IAZA or FAZA was carried out as described earlier. Stained cells were resuspended in 150 μL of methanol and the optical density (OD) was measured at 584 nm using a FLUOstar OPTIMA microplate reader (BMG Labtech, Ortenberg, Germany). The percentage of attached cells was calculated by subtracting OD of blank wells and then normalizing DMSO controls (under respective O_2_ levels) to 100%. The first point on each curve represents 0 μM drug.

### 2.4 Clonogenic survival assay

FaDu cells seeded in 60 mm glass petri dishes (at densities ranging from 300 to 1500 cells per plate) were treated with different concentrations of IAZA or FAZA (up to 1000 μM) for 24 h under normoxia or hypoxia (1% O_2_, 0.1% O_2_ and <0.1% O_2_); 0.02% DMSO was used as vehicle control. After treatment, hypoxic cells were reoxygenated, medium was changed, and cells were allowed to grow in drug-free medium for 14 days. For analysis of adhesion properties, FaDu cells (8 × 10^5^) treated with drugs (100 μM) or vehicle control for 24 h under normoxia or hypoxia (<0.1% O_2_) were trypsinized, replated at low seeding densities (300–600 cells per 60 mm plate) and allowed to grow and form colonies for 14 days. Colonies were stained with crystal violet and counted manually. Colony counts were converted to percentage of seeding densities, and then normalized to vehicle-treated controls under respective O_2_ conditions.

### 2.5 Apoptosis assay

FaDu cells (8 × 10^5^) were seeded on 60-mm glass petri dishes and allowed to attach overnight. Cells were then treated with either 0.02% DMSO or drug (100 μM IAZA or 100 μM FAZA) and incubated under normoxia and hypoxia (<0.1% O_2_) for 24 h, followed by incubation under normoxia for 48 h. Afterwards, cells were trypsinized, counted and 1 × 10^6^ cells per condition were stained with annexin V and propidium iodide (640914, BioLegend, San Diego, CA). Stained cells were analyzed using an Attune® NxT Acoustic Focusing Cytometer (Thermo Fisher Scientific, Waltham, MA); 100,000 events were recorded for each condition. CPT-treated cells (20 nM, 24 h) served as positive control for apoptosis.

### 2.6 Senescence assay

After treating FaDu cells with either 0.02% DMSO, 100 μM IAZA or 100 μM FAZA under normoxia and hypoxia (<0.1% O_2_) for 24 h, drug containing medium was replaced with fresh medium, and cells were allowed to recover and grow for 4 days. To detect senescence, cells were stained with a beta-galactosidase staining kit following the manufacturer's protocol (9860, Cell Signalling Technology, Danvers, MA). Cells treated with 8 Gy ionizing radiation were used as positive control for senescence. Images were obtained with a Zeiss Axioscope colour microscope (Carl Zeiss, Jena, Germany).

### 2.7 Cell counting assay

FaDu cells (1 × 10^5^) seeded on 60-mm glass petri dishes were left overnight to attach, after which they were treated with either vehicle control (0.02% DMSO) or drug (100 μM IAZA or 100 μM FAZA) and incubated under normoxia and hypoxia (<0.1% O_2_) for 24 h. Following treatment, cells were either immediately trypsinized and counted with a Beckman Coulter Z2 Particle Counter (Beckman Coulter, Brea, CA), or allowed to grow in drug free medium under normoxia. Subsequently, cells were trypsinized and counted every other day for up to 12 additional days.

### 2.8 Cell cycle profile analysis

FaDu cells treated with drug (IAZA or FAZA, 100 μM) or vehicle control (0.02% DMSO) under normoxia or hypoxia (<0.1% O_2_) for 24 h were either immediately trypsinized after treatment, or allowed to recover in drug free medium under normoxia for up to 24 h. Trypsinized cells were counted and 1 × 10^6^ cells were fixed in 70% ethanol, washed with cold phosphate-buffered saline (PBS) and incubated with 100 μg/ml RNase A (1007885, QIAGEN, Hilden, Germany) for 10 min at room temperature (∼22 °C). Cells were then washed and mixed with propidium iodide (PI; P4170, Sigma-Aldrich) to a final concentration of 50 μg/ml to stain their DNA content. Processed samples were analyzed using a BD FACSCanto II flow cytometer (BD Biosciences, Franklin Lakes, NJ) to detect PI fluorescence upon gating for forward and side scatter; singlet population was chosen by gating for PI-width and PI-area.

### 2.9 Click-iT EdU incorporation assay

To evaluate the effects of IAZA and FAZA on DNA synthesis, FaDu cells grown on sterilized glass coverslips were incubated with drug (100 μM) or vehicle control (0.02% DMSO) in medium containing 10 μM 5-ethynyl-2′-deoxyuridine (EdU, C10339, Invitrogen, Waltham, MA) for 24 h under normoxia or hypoxia (<0.1% O_2_), followed by fixation in 2% paraformaldehyde (PFA, P6148, Sigma-Aldrich). To assess the capacity of drug-treated cells to re-initiate DNA synthesis, FaDu cells treated with IAZA or FAZA (or 0.02% DMSO) for 24 h under normoxia and hypoxia (<0.1% O_2_) were allowed to recover for 24 h in drug-free medium containing 10 μM EdU, and then fixed in 2% PFA. Fixed cells were processed for “click” staining according to the manufacturer's protocol (C10339, Invitrogen); nuclei were counterstained with Hoechst (Life Technologies, Carlsbad, CA). Coverslips were mounted on glass slides (Fluoroshield Mounting Medium, Abcam, Cambridge, UK) and imaged with a Plan-Apochromat 40X/1.3 Oil DIC lens on a ZEISS 710 confocal microscope using Zen 2011 software (Carl Zeiss, Jena Germany).

### 2.10 DNA fibre assay

The effect of drugs on DNA replication was measured by a DNA fibre assay as previously described with some modifications [21]. Briefly, cells were incubated with 5-iodo-2′-deoxyuridine (33 μM; IdU; I7756, Sigma-Aldrich) for 30 min followed by several PBS washes. Cells were then incubated with 500 μM drug (or 0.02% DMSO) under normoxia or hypoxia (<0.1% O_2_) for 3 h. Afterwards, drug-containing medium was removed, cells were washed several times with PBS, and were allowed to recover for 30 min in fresh medium containing 5-chloro-2′-deoxyuridine (250 μM; CldU; C6891, Sigma-Aldrich). Following incubation with CldU, cells were trypsinized and harvested through centrifugation. The cell pellet was then resuspended in PBS at 100,000 cells/ml; 2 μl of the cell suspension was spotted on a positive charged glass slide. The cell suspension drop was mixed with 10 μl of DNA fiber lysis buffer (200 mM Tris–HCl pH 7.5, 50 mM EDTA, 0.5% SDS) and DNA strands were allowed to migrate down the slides by tilting the slides at a 15° angle. The resulting DNA spreads were then air dried for 40 min followed by a fixation in 3:1 methanol/acetic acid for 5 min. A 30 min incubation with 2.5 M hydrochloric acid was used to denature DNA fibers, then slides were washed with PBS and blocked for 1 h in 5% bovine serum albumin (BSA; A9647, Sigma-Aldrich) in PBS-0.1% Tween-20. DNA immunostaining was performed with a rat anti-BrdU/CldU antibody to detect CldU (1:200 dilution; 347580, BD Biosciences) and a mouse anti-BrdU/IdU antibody to detect IdU (1:200 dilution; ab6326, Abcam) in a humidified chamber for 1 h at room temperature. Secondary antibodies used include anti-rat-Alexa Fluor 488 (1:200 dilution; A-21470, Thermo Fisher Scientific) and goat anti-mouse-Alexa Fluor 546 (1:200 dilution; A-21123, Thermo Fisher Scientific). Slides were mounted in ProLong™ Gold Antifade Mountant (P10144, Thermo Fisher Scientific), and imaged with an upright fluorescence microscope (ZEISS AxioImager.Z1). More than 150 fibers were analyzed for each data set.

### 2.11 Trapped in agarose DNA click staining (TARDCS)

To assess covalent drug-DNA binding, we modified a previously described trapped in agarose DNA immunostaining (TARDIS) assay [22]. Instead of antibody based detection, our method utilizes click chemistry by using a click chemistry compatible 2-NI compound, N_3_-AZA (Fig. 1C) [20]. Briefly, FaDu cells treated with 100 μM N_3_-AZA (or 0.02% DMSO) under normoxia or hypoxia (<0.1% O_2_) for 24 h were harvested by trypsinization and pelleted by centrifugation. The cell pellet was then mixed with 1% low melting point agarose and spread on glass slides pre-coated with 1% normal agarose. Slides were processed as described in Ref. [22] by placing them in lysis buffer for 30 min followed by a 30 min incubation in 1 M NaCl. Slides were then air dried, select slides were treated with either 1000 U DNase I (04536282001, Roche, Basel, Switzerland) or 0.2 mg proteinase K (19131, QIAGEN). Afterwards, slides were blocked in 1% BSA for 20 min and click staining was performed with an Alexa Fluor 594 alkyne (A10275, 1:5000 dilution, Molecular Probes, Eugene, OR) using a Click-iT™ Cell Reaction Buffer Kit (C10269, Thermo Fisher Scientific); DNA was counterstained with Hoechst. Slides were imaged with a Plan-Apochromat 20X/0.8 M27 lens on a Zeiss 710 confocal microscope using Zen 2011 software.

### 2.12 Immunocytochemistry

FaDu cells grown on sterilized glass coverslips were treated with vehicle control or drug (100 μM) for 24 h under normoxia or hypoxia (<0.1% O_2_), after which cells were fixed in 2% PFA (or methanol), blocked in 1% BSA for 20 min, and probed with the following primary antibodies: anti-phospho-histone H2A.X (Ser139) (γ-H2AX; 1:5000 dilution, 05–636, Millipore Sigma, Burlington, MA), anti-replication protein A2 (RPA; 1:2500 dilution, ab2175, Abcam) or anti-proliferating cell nuclear antigen (PCNA; 1:250 dilution, ab29, Abcam). Secondary antibodies used include Alexa Fluor 488 conjugated anti-rabbit IgG (1:1000 dilution; Molecular Probes) and Alexa Fluor 594 conjugated anti-mouse IgG (1:250 to 1:1000 dilution; Molecular Probes). Cells processed for RPA were first incubated in extraction buffer (25 mM HEPES, 300 mM sucrose, 50 mM NaCl, 1 mM EDTA, 3 mM MgCl_2_, 0.5% NP-40, pH 7.9) for 3 min prior to fixation in PFA. For chromatin bound PCNA detection, cells underwent a detergent extraction step in ice-cold hypotonic buffer (10 mM HEPES-KOH at pH 7.9, 10 mM KC1, 1.5 mM MgC1_2_, 0.5 mM DTT, 0.1% Triton) for 10 min before they were fixed in cold methanol. Irradiated (5 Gy) cells and cells treated with 5 mM HU (24 h) served as positive controls for γ-H2AX and RPA staining, respectively. To label actin microfilaments, fixed cells were stained with Phalloidin-iFluor 488 reagent (1:1000 dilution, ab176753, Abcam). Nuclei were counterstained with Hoechst and coverslips were mounted on glass slides. Images were obtained with a Plan-Apochromat 40X/1.3 Oil DIC lens on a Zeiss 710 confocal microscope using Zen 2011 software.

### 2.13 Alkaline comet assay

FaDu cells were treated with drugs (100 μM) or vehicle control for 24 h under normoxia and hypoxia (<0.1% O_2_); cells exposed to 5 Gy ionizing radiation were used as positive controls. Immediately after irradiation, cells were harvested in PBS by scraping, mixed with low melting point agarose, and spread on comet slides. After the gel solidified, slides were put in lysis solution (4250-050-01, R&D Systems, Minneapolis, MN) for 1 h, and then in unwinding solution (200 mM NaOH, 2.5 mM EDTA) for 1 h at 4 °C. Slides were then placed in alkaline electrophoresis buffer (200 mM NaOH, 1 mM EDTA), and current was applied at 21 V for 45 min. Afterwards, slides were washed twice in distilled H_2_O, fixed with 70% ethanol for 5 min, and air dried before staining DNA with ethidium bromide (E7637, Sigma-Aldrich). Slides were imaged with an upright fluorescence microscope (Zeiss AxioImager.Z1), and comets were analyzed using CometScore™ software (TriTek Corp., Sumerduck, VA).

### 2.14 Assessing hydrogen peroxide (H_2_O_2_) levels

The ROS-Glo™ H_2_O_2_ assay kit (G8820, Promega, Madison, WI) was used to analyze the levels of H_2_O_2_ as a readout of the reactive oxygen species (ROS) generated in response to drug treatment. Briefly, FaDu cells seeded in 96-well plates were treated with IAZA (100 μM and 150 μM), FAZA (100 μM and 350 μM) or 0.02% DMSO for 72 h. Cells were then taken out of the hypoxia chamber and the H_2_O_2_ substrate was added, which generates a luciferin precursor upon reaction with H_2_O_2_. Cells were incubated with the substrate for 6 h, either under normal O_2_ conditions (reoxygenation) or placed back in the hypoxia chamber. Afterwards, 50 μl of medium from each well was mixed with equal volume of ROS-Glo detection solution in a separate plate, incubated for 20 min, and the luciferin luminescence (which is directly proportional to the amount of H_2_O_2_ level present in the reaction well) was recorded using an OPTIMA microplate reader. Cells in the original sample plate were assayed for viability using crystal violet staining. Luminescence data were normalized by the cell viability results.

### 2.15 Assessing cell viability and metabolic activity in drug-treated cells following replating

FaDu cells (8 × 10^5^) treated with drugs (100 μM) or vehicle control for 24 h under normoxia or hypoxia (<0.1% O_2_) were trypsinized, replated in 96-well plates (5000 cells/well), and allowed to grow for 3 days. Cells were then stained with LIVE/DEAD™ Cell Imaging Kit (R37601, Thermo Fisher Scientific) and imaged with a Plan-Apochromat 20x/0.8 M27 lens on a Zeiss 710 confocal microscope. Viable cells are identified by their esterase activity that converts non-fluorescent cell-permeant calcein AM to the intensely fluorescent calcein (green, labelled as “live”), while the compromised membranes of dead cells allow binding of BOBO-3 Iodide to the DNA (red). To measure cellular metabolic capacity, 50 μl of 3-(4,5-dimethylthiazol-2-yl)-2,5-diphenyltetrazolium bromide (MTT; 11465007001, Millipore Sigma) was added to each well, incubated for 3 h, and imaged as mentioned before. Cellular capacity to form formazan crystals was analyzed as a readout of metabolic activity.

### 2.16 Immunoblotting

FaDu cells treated with 100 μM IAZA or FAZA (or 0.02% DMSO) for 24 h under normoxia and hypoxia (<0.1% O_2_) were harvested in RIPA buffer supplemented with protease inhibitor. 50 μg of crude protein extracts were separated on a 10% polyacrylamide gel for 55–70 min at 180 V, transferred onto nitrocellulose membranes and blocked for 1 h with 5% milk (in PBS, 0.1% Triton-100). Total protein was visualized using Revert™ 700 Total Protein Stain (926–11016, LI-COR, Lincoln, NE). Membranes were probed with the following primary antibodies: anti-hypoxia inducible factor 1 alpha (1:2000 dilution; NB100-449, Novus Biologicals, Littleton, CO), anti-glyceraldehyde-3-phosphate dehydrogenase (1:1000 dilution, ab9482, Abcam), anti-glutathione S-transferase P (1:2000 dilution, ABS1650, Millipore Sigma), anti-Cyclin E1 (1:1000 dilution, ab133266, Abcam), anti-beta tubulin (1:4000 dilution; ab6046, Abcam) and anti-beta actin (1:2000 dilution; sc-47778, Santa Cruz Biotechnology, Dallas, TX). Secondary antibodies used include goat anti-rabbit-IgG-HRP (1:2000 dilution; Jackson Immunoresearch, West Grove, PA), IR 800 conjugated goat anti-mouse IgG (1:2000 dilution, 926–80010, LI-COR) and IR 800 conjugated goat anti-rabbit IgG (1:2000 dilution; 926–32211, LI-COR). Membranes were then processed with West Pico PLUS chemiluminescent substrate (Thermo Fisher Scientific) and scanned with an Odyssey Fc imager (LI-COR).

### 2.17 Glyceraldehyde-3-phosphate dehydrogenase (GAPDH) activity assay

FaDu cells treated with IAZA (150 μM), FAZA (350 μM) or vehicle control (0.02% DMSO) for 24 h under normoxia or hypoxia (<0.1% O_2_) were harvested by trypsinization and pelleted by centrifugation. Cellular GAPDH enzyme activity was quantified as described earlier using a GAPDH activity assay kit (ab204732, Abcam) [20].

### 2.18 Glutathione S-transferase (GST) assay

Total cellular GST enzymatic activity was measured using a GST assay kit (CS0410, Sigma-Aldrich). FaDu cells treated with IAZA (100 μM and 150 μM), FAZA (100 μM and 350 μM) or vehicle control (0.02% DMSO) for 24 h under normoxia or hypoxia (<0.1% O_2_) were processed to quantify total GST activity as previously described [20].

### 2.19 Total glutathione (GSH) quantification

Total cellular GSH levels were measured with DTNB [5,5′-dithio-*bis*-2-(nitrobenzoic acid)] that reacts with GSH and produces yellow colored TNB (5-thio-2-nitrobenzoic acid); the rate of TNB production is directly proportional to the concentration of GSH in the sample (703002, Cayman Chemical, Ann Arbor, MI). Briefly, FaDu cells treated with IAZA and FAZA at indicated concentrations for 24 h under normoxia or hypoxia (<0.1%O_2_) were harvested by scraping, counted, and pelleted by centrifugation. The cell pellet was dissolved in PBS containing 2 mM EDTA (1 × 10^6^ cells in 100 μl), sonicated and centrifuged again. The supernatant was deproteinated using metaphosphoric acid (239275, Sigma-Aldrich) and mixed with 4 M triethanolamine (T58300, Sigma-Aldrich) as per the manufacturer's guidelines. The resultant samples were used to perform the total glutathione assay in a 96-well plate. A standard curve was generated using the GSH standard provided with the assay kit, and concentrations of GSH in samples were determined from the standard curve.

### 2.20 RNAi transfection

FaDu cells seeded in 6-well plates (∼4 × 10^5^ cells per well) were transfected either with control non-targeting siRNA (50 nM, D-001810-10-05; GE Healthcare Dharmacon, CO, USA) or siRNA targeting GAPDH (50 nM, L-004253-00-0005, GE Healthcare Dharmacon), GSTP1 (50 nM, L-011179-00-0005, GE Healthcare Dharmacon) or both GAPDH and GSTP1 siRNAs for ∼18 h using Lipofectamine® RNAiMAX reagent (13778–075, Life Technologies, CA, USA). Afterwards, cells were allowed to grow in fresh medium for an additional 72 h, harvested in RIPA buffer and processed for immunoblotting. For viability analysis, FaDu cells seeded in 96-well plates (12,000 cells per well) were transfected as mentioned above and cell viability was assessed by staining cells with crystal violet.

### 2.21 *In vivo* toxicity assay

Animal experiments were carried out according to the protocols approved by the Cross Cancer Institute Animal Care Committee (protocol # AC18239) in accord with the Canadian Council on Animal Care guidelines. To establish the toxicity profile of intraperitoneal (i.p.) administered IAZA, NOD/SCID/IL2R mice were injected with vehicle control (15% DMSO v/v in 150 μl sterile water) or with 3 different IAZA doses (200, 400 or 600 mg/kg body weight, b.w.). Mice were monitored for weight loss, responsiveness and body conditioning for 14 days, after which, animals were euthanized. During the post-mortem examination, blood was collected via heart puncture, and processed for blood chemistry (samples analyzed at IDEXX laboratory, Edmonton, AB). Additionally, a set of organs (brain, heart, kidney, liver and lung) was removed from each animal for pathological analysis. These were prepared by fixation in 10% neutral buffered formalin for a minimum of 24 h. Following fixation, tissues were trimmed with a scalpel to a thickness of 2–3 mm and a section of each organ was placed in a tissue cassette. Tissues in cassettes were processed into paraffin, embedded in a paraffin block, sectioned on a microtome to a thickness of 5 μm, placed on a microscope slide and stained with hematoxylin and eosin stain, all procedures following standard histology techniques. Slides were examined by a board-certified veterinary pathologist using a Nikon Eclipse 80i microscope and observations recorded for each section. Photomicrographs were taken using a Moticam 10 digital camera.

### 2.22 Tumour growth delay assay and tissue staining

For tumour growth control experiments, NU/NU nude mice were injected with ∼6 × 10^6^ FaDu cells subcutaneously on the upper right flank. Tumours were measured with a Vernier caliper, and volume was determined by the formula: *(π/6)ab*^*2*^, where *a* is the longest and *b* is the perpendicular shorter tumour axis. Once the tumours reached a volume between ∼200 and 300 mm^3^, mice were injected i.p. with either vehicle control (up to 15% DMSO v/v in 150 μl sterile water) or IAZA (400 mg/kg b.w. in 150 μl DMSO: sterile water). Mice were monitored daily following treatment, and were sacrificed when tumours reached a volume of ∼1500 mm^3^. 2 h prior to sacrifice, mice were injected i.p. with N_3_-AZA (60 mg/kg b.w.). After euthanization, tumours were excised, flash frozen in optimal cutting temperature compound (O.C.T., 4585, Fisher Scientific, Waltham, MA), and N_3_-AZA click chemistry was performed on central sagittal tumour sections to determine endpoint hypoxia levels as described previously [20]. Briefly, acetone-fixed frozen tumour sections were blocked in PBS with 1% BSA, incubated with primary antibody for small vessel endothelium (rat anti-CD31, 1:50 dilution; 550274, BD Pharmingen) for 2 h, followed by incubation with the secondary antibody (Alexa Fluor 647 goat anti-rat IgG, 1:1000 dilution; Molecular Probes) mixed with 4′,6-diamidino-2-phenylindole (DAPI). Afterwards, tumour sections were incubated with the Click-IT reaction cocktail (Thermo Fisher Scientific) for 30 min, which was made up according to the manufacturer's directions using a 1:5000 dilution of the 2 mg/mL Alexafluor 555-conjugated alkyne stock (A20013, Molecular Probes). After washing, sections were mounted using Mowiol 4–88 mounting media (Millipore, Etobicoke, ON), and images were obtained by tile scan with a Leica SP8 STED microscope (Leica Microsystems GmbH, Wetzlar, Germany). An estimate of hypoxic tumour fraction was calculated with MATLAB R2018b (MATLAB and Image Processing Toolbox Release 2018b, The MathWorks, Inc., Natick, Massachusetts, United States) as described before [20]. Images were converted to binary by applying a manual threshold (determined from the background tissue staining) to the N_3_-AZA channel, while the Otsu threshold was applied to the DAPI channel [23]. The hypoxic fraction estimate was calculated using the following formula:

$$\text{Hypoxic fraction }\left(\text{HF}\right)=\frac{\mathrm{Area}\;\mathrm{of}\;\mathrm{pixels}\;\mathrm{stained}\;\mathrm{with}\;N3-\mathrm{AZA}}{\mathrm{Area}\;\mathrm{of}\;\mathrm{pixels}\;\mathrm{stained}\;\mathrm{with}\;\mathrm{DAPI}}\text{*}100$$

### 2.23 Data processing and statistics

Adobe Photoshop (Adobe Inc., San Jose, CA) was used to adjust for brightness and contrast, to add scale bars, and for rearrangement and labelling in immunoblots and microscopic images. Gel scans were quantified using Image Studio lite v5.2 (LI-COR Biosciences, Lincoln, NE); protein band values were divided by the total protein stain (or loading control) for respective lanes and normalized to vehicle-treated normoxia controls. EdU click staining, γ-H2AX, RPA and PCNA stained microscopic images were processed with IMARIS software (Bitplane, Zürich, Switzerland) to quantify click/antibody staining intensity within the nucleus using Hoechst staining as a nuclear mask. Histograms were generated with intervals of staining intensity on the x axis and the percentage of cells with intensity levels that fall within that range on the y axis. For γ-H2AX data analysis, an arbitrary threshold was chosen such that the γ-H2AX staining intensity of ∼95% of cells in the vehicle-treated normoxic sample fell below this threshold. The percentage of cells above this threshold was considered positive for γ-H2AX. To identify chromatin-bound PCNA positive cells, PCNA staining in mitotic cells was used as threshold for background staining. DNA fibre tract lengths were measured with MetaMorph software (Molecular Devices, San Jose, CA) to convert the pixel values to micrometers and plotted as scatterplots. Graph generation and statistical analysis were performed using GraphPad Prism V7 (GraphPad Software, La Jolla, CA). IC_50_ values from the crystal violet staining and colony formation assays were generated using non-linear regression analysis. Graphs display the mean with standard error of the mean (S.E.M.). The 2-tailed unpaired *t*-test was used for statistical analysis, with p < 0.05 considered statistically significant. GAPDH activity was analyzed using Dunnett's 2-way ANOVA. Asterisks depict statistically significant differences: ns (not significant), * (P ≤ 0.05), ** (P ≤ 0.01), *** (P < 0.001), **** (P < 0.0001).

## 3 Results

### 3.1 IAZA and FAZA selectively affect viability and clonogenicity of O_2_ starved cells

Experiments were carried out primarily with a human head and neck squamous cell carcinoma cell line, FaDu. Additional cell lines used for validation purposes included A549 (human lung epithelial carcinoma), PC3 (human prostate carcinoma) and HCT116 (human colorectal carcinoma). Sensitivity of FaDu cells to IAZA and FAZA treatment under different O_2_ levels was determined by crystal violet staining (Fig. 1D and E) and clonogenic survival assays (Fig. 1G and H); effects of O_2_ levels on FaDu cell viability and clonogenicity are shown in Fig. 1F and I, respectively. Hypoxic cells displayed considerably greater sensitivity to both compounds than normoxic cells, with IAZA showing a more potent response than FAZA at the same concentrations. In the colony formation assay, IAZA and FAZA showed the strongest effects on cells cultured under <0.1% O_2_, with IC_50_ values > 22-fold (IAZA) or >15-fold (FAZA) lower than that obtained under normoxia (Fig. 1F and G). A similar trend was seen in other cell lines used in this study as well, with hypoxic (0.1% O_2_) cells displaying ∼4–12 fold (IAZA) or ∼7–12 fold (FAZA) more sensitivity to the drugs than their normoxic counterparts (Fig. S1), regardless of their p53 status (FaDu and PC3 express mutated p53, while A549 and HCT116 have wild-type p53). In subsequent *in vitro* experiments assessing their therapeutic efficacy, a drug concentration of 100 μM was used, except for the DNA fibre assay (500 μM), ROS quantification (hypoxic IC_50_ concentrations) and enzymatic assays (hypoxic IC_50_ concentrations).

### 3.2 IAZA and FAZA treatment under hypoxia do not increase cell death or senescence, but affect cell proliferation

**Fig. 2:**
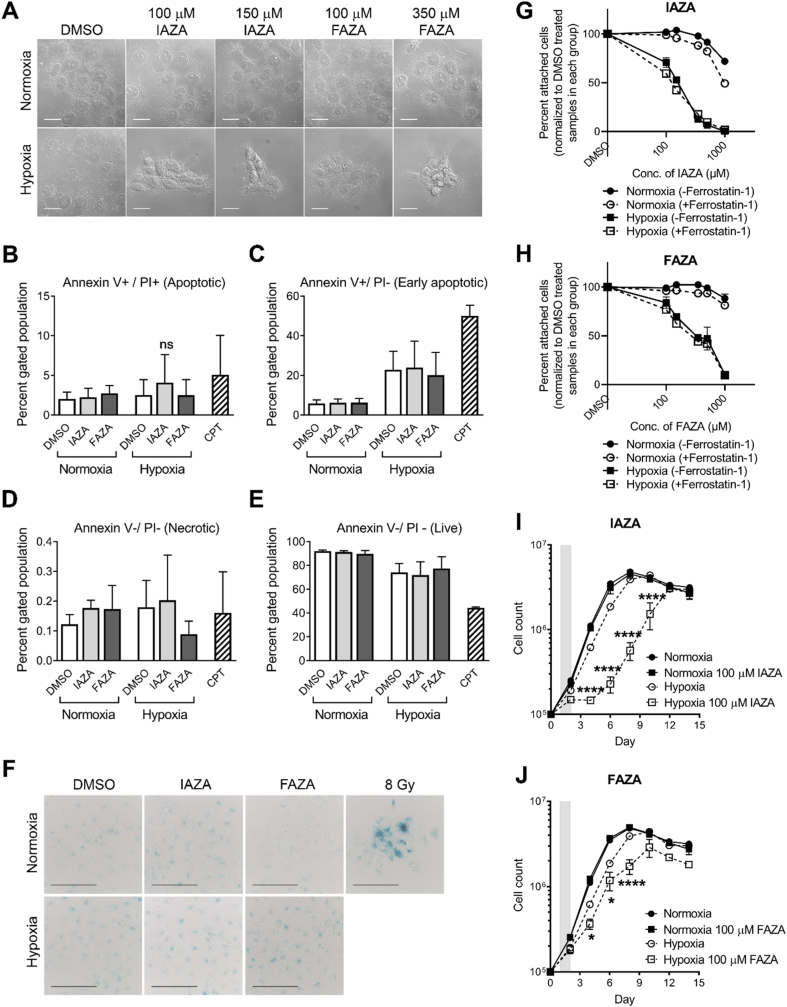
**IAZA and FAZA treatment do not induce apoptosis, senescence or ferroptosis under hypoxia, but reduce proliferation in hypoxic/reoxygenated cells.** IAZA and FAZA treatment induced morphological changes in a dose dependent manner, but only under hypoxia (**A**). Compared to vehicle control, hypoxic/reoxygenated drug-treated cells did not show a statistically significant change in apoptotic (**B**), early apoptotic (**C**), necrotic (**D**) or live population (**E**). IAZA- and FAZA-treated hypoxic/reoxygenated cells stained poorly for beta-galactosidase activity (**F**). Addition of Ferrostatin-1 did not change the toxicity profile of IAZA (**G**) or FAZA (**H**). IAZA- or FAZA-treated hypoxic/reoxygenated cells show slower proliferation rates (**I** and **J**); shaded regions show duration of drug exposure. Data represent mean ± S.E.M; scale bar = 20 μm (**A**) and 100 μm (**F**).

While standard cytotoxicity assays provide valuable insights into cells’ sensitivity towards a particular treatment, they do not necessarily capture treatment-induced cellular fate [24]. For example, the CVS assay measures relative attached (hence viable) cells; a decrease in which may result from either increased cell death or from induction of senescence or cytostasis [25]. Similarly, recent observations with clonogenic survival assays identified certain dormant cell populations that can remain viable and metabolically active without forming macroscopic colonies [26]. Hence, additional experiments are required to properly characterize cellular fate induced by a treatment. Morphologically, IAZA- and FAZA-treated hypoxic cells appeared more compact and stacked together, with a marked shrinkage of their cytoplasm (Fig. 2A). To determine if these 2-NI compounds induced cell death (apoptosis or necrosis) under hypoxia, FaDu cells treated with drugs (or vehicle control) were stained with annexin V and PI, and analyzed by flow cytometry. No significant increase in apoptotic or necrotic populations were observed in drug-treated hypoxic samples; most cells stained negative for annexin V and PI (alive) (Fig. 2B–E). Next, we checked if hypoxic cells underwent irreversible growth arrest (senescence) when treated with IAZA or FAZA. Senescent cells display increased lysosomal biogenesis, resulting in a characteristic beta-galactosidase (β-gal) activity detectable at pH 6.0 (termed ‘senescence-associated beta-galactosidase’ activity), which can be used to distinguish senescent cells within a population [27]. Staining FaDu cells treated with IAZA and FAZA for beta-galactosidase activity revealed no difference between vehicle and drug-treated hypoxic cells, implying that these drugs do not induce senescence (Fig. 2F). Recently, 2-NIs (at millimolar range) were reported to induce ferroptosis in hypoxic glioma stem cells [28]. Therefore, induction of ferroptosis was assessed by treating cells with IAZA/FAZA in the presence or absence of Ferrostatin-1 (a ferroptosis inhibitor), which protects cells from ferroptosis inducing agents. Interestingly, addition of Ferrostatin-1 did not improve viability of IAZA/FAZA-treated hypoxic cells, thus ruling out ferroptosis as a consequence of drug treatment (Fig. 2G and H). Since no induction of cell death or senescence was observed in IAZA- or FAZA-treated hypoxic cells, we next monitored changes in cell proliferation rates in response to drug treatment, and after fixed periods of recovery (and reoxygenation). Hypoxic incubation with IAZA or FAZA (100 μM) decreased total cell counts when compared to vehicle-treated hypoxic cells; no effects were seen under normoxia (Fig. 2I and J). Drug-induced inhibitory effects on hypoxic/reoxygenated cell growth were not immediately alleviated by drug removal and reoxygenation, which was evident from the sustained slower proliferation rates during the recovery period. Since FAZA was used at a concentration ∼3 times lower than its hypoxic IC_50_ value, it generated a milder response in comparison to IAZA.

### 3.3 Hypoxic treatment with IAZA or FAZA slows down cell cycle progression, represses DNA synthesis, and induces replication stress

**Fig. 3:**
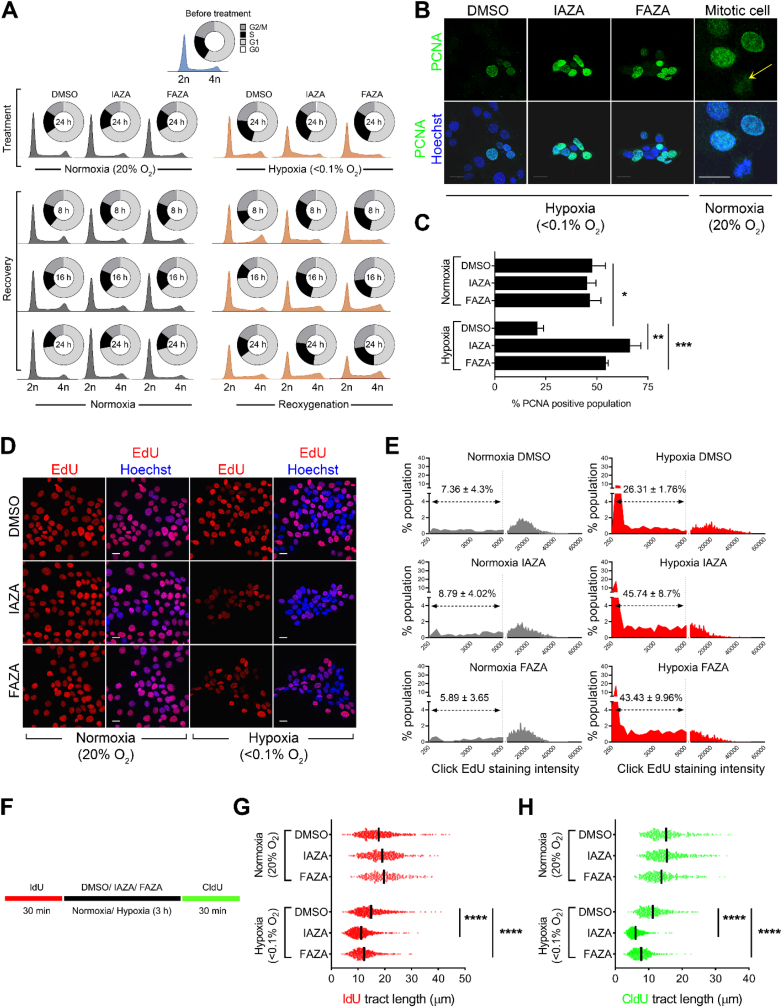
**Hypoxic cells treated with IAZA and FAZA show slower S phase progression, increased chromatin-bound PCNA staining, reduced DNA synthesis, and signs of replication stress.** Flow cytometric analysis for cell cycle phases showed an increase in S phase population in IAZA- and FAZA-treated hypoxic cells. These cells continuously showed slower S phase progression, even when drugs were removed, and hypoxic cells were allowed to reoxygenate (**A**). Hypoxic cells, when treated with IAZA and FAZA, showed increased chromatin bound PCNA staining, which is significantly higher than vehicle-treated hypoxic cells (**B** and **C**); yellow arrow shows mitotic cell (**B**). Hypoxic cells treated with IAZA and FAZA displayed reduced EdU incorporation (**D**). Intensity of EdU click-stained micrographs was quantified and plotted as intensity versus %population histograms. Hypoxic exposure by itself increased cell population with low EdU staining; incubation with IAZA and FAZA under hypoxia further increased “low EdU stained” cell fractions (**E**). Experimental setup for DNA fibre assay (**F**). IAZA- and FAZA-treated cells showed nascent fork degradation (**G**) and diminished ability to restart replication (**H**) in the DNA fibre assay. Representative micrographs are shown; scale bar = 20 μm (**B** and **D)**. Quantification shows mean ± S.E.M. from three independent experiments. (For interpretation of the references to color in this figure legend, the reader is referred to the Web version of this article.)

Given that IAZA and FAZA treatment negatively affected cell proliferation under hypoxia, their impacts on cell cycle distribution were analyzed using PI-flow cytometry. IAZA- and FAZA-treated hypoxic cells showed a significant increase in the S phase population (Figs. 3A and S2A). Time-course analysis of cell cycle progression revealed that drug-treated hypoxic cells progressed through S phase at a slower pace, even after treatment had ended (Fig. 3A). A significant increase in the chromatin bound PCNA-positive population (Fig. 3B and C) was seen in hypoxic cells treated with IAZA/FAZA, which is reflective of late G1 and early S phase cells [29]. Moreover, when compared to vehicle-treated hypoxic cells, IAZA- and FAZA-treated hypoxic cells showed higher cyclin E1 levels (Figs. S2B and C), a cell cycle protein upregulated during G1/S transition [30]. To identify why cells were stalling at S phase in response to 2-NI treatment under hypoxia, DNA replication efficiency was analyzed by monitoring the cellular uptake of EdU. EdU is a nucleoside analogue that gets incorporated into cellular DNA during replication; prolonged incubation with EdU can thereby be used to distinguish between replication proficient and replication deficient cells. Hypoxic cells treated with IAZA or FAZA (100 μM) showed reduced EdU uptake, which is indicative of compromised DNA synthesis (Fig. 3D). Quantification of cellular EdU staining, presented as histograms (Fig. 3E), revealed that 24 h hypoxic exposure by itself affected EdU uptake in cells; addition of IAZA and FAZA under hypoxia further slowed down DNA replication rates (evident from a significant increase in cell fraction with low EdU). Moreover, replication was still compromised in hypoxic drug-treated cells that were allowed to reoxygenate and recover in drug-free medium (containing EdU) (Fig. S2D).

Slower rates of DNA replication are known to cause replication stress, which can be determined by staining cells for replication protein A (RPA) [31]. RPA binds to single-stranded DNA generated as an intermediate from stalled replication forks, or during recombination-mediated repair of DNA damage [32]. Chromatin-bound RPA resists detergent extraction that removes the nucleoplasmic and cytoplasmic proteins. Higher chromatin retention of RPA was seen in drug-treated hypoxic cells, which is suggestive of increased ssDNA. However, the effect was not as severe as seen in cells treated with hydroxyurea (5 mM), a potent DNA replication inhibitor (Figs. S2E and F). In response to replication stress, the replication fork also undergoes fork reversal for maintaining genome stability, which can be assessed through the DNA fibre assay [33]. Nucleolytic degradation following replication fork stalling was monitored by pulse-labeling cells with two different thymidine analogs (IdU and CldU), with a 3 h drug treatment (under normoxia or hypoxia) in between the two labelling steps (Fig. 3F). Shortening of the first tract serves as a readout of nascent fork degradation, which in turn reflects induction of replication stress [33]. Only forks characterized by contiguous IdU-CldU signals (and not on forks that have only the IdU label) were analyzed to ensure that the shortening phenotype is indeed due to nucleolytic degradation of stalled replication forks, and not because of premature termination events [34]. Quantification of DNA fibres revealed a significant decrease in IdU track lengths (Fig. 3G). CldU track lengths were also significantly shortened, which points towards an inability of drug-treated hypoxic cells to restart replication after reoxygenation (Fig. 3H). Taken together, these observations suggest that IAZA and FAZA, at concentrations used in these experiments, induced replication stress under hypoxia by compromising DNA synthesis and consequently slowed down cell cycle progression.

### 3.4 IAZA- and FAZA-treated hypoxic cells show higher γ-H2AX signal

**Fig. 4:**
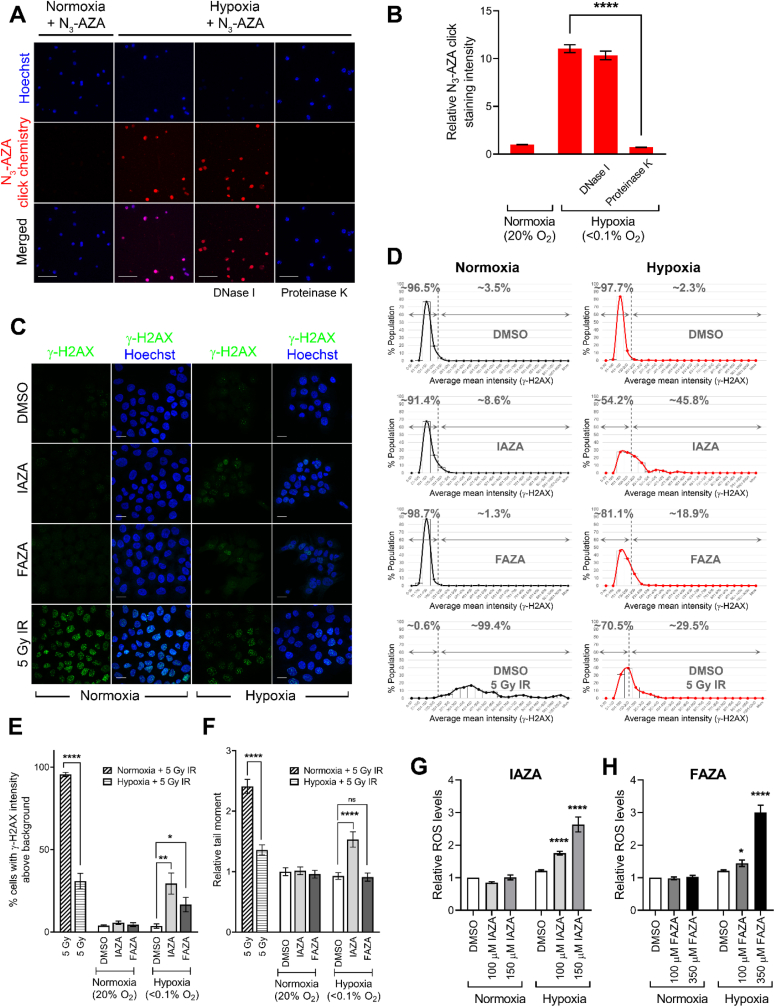
**Analysis of DNA-NI binding characteristics and NI effects on DNA integrity under hypoxia.** Trapped in agarose DNA click staining (TARDCS) assay revealed proteins, rather than DNA, form covalent adducts with NIs (**A**). Quantification of N_3_-AZA click staining in TARDCS assays (**B**). FaDu cells treated with DMSO, IAZA and FAZA under normoxia and hypoxia (<0.1% O_2_) were processed for γ-H2AX immunostaining (**C**). Quantifications of γ-H2AX immunostaining data are represented as histograms (**D**). A statistically significant increase in percent population with γ-H2AX staining higher than background was seen in hypoxic drug-treated cells (**E**). Alkaline comet assay showed a significant increase in comet tail moment only in IAZA-treated hypoxic cells (**F**). A significant increase in H_2_O_2_ levels was found in IAZA (**G**) and FAZA (**H**) treated hypoxic cells upon reoxygenation. Representative micrographs are shown; scale bar = 100 μm (**A**) and 20 μm (**C**). Quantification shows mean ± S.E.M. from at least three independent experiments.

DNA has often been cited as a major site of attack by activated NIs [35]. It has long been argued that activated NIs can directly bind to DNA and induce strand breaks [36,37]. To characterize the nature of DNA-NI interaction, we performed a modified TARDIS assay using N_3_-AZA, which is an azido analogue of IAZA and FAZA (Fig. 1C). N_3_-AZA allows for direct detection of NI-adducts with fluorescent alkynes in a Cu(I)-catalyzed click chemistry reaction [20]. Similar to the standard TARDIS assay, the modified assay (hereafter referred to as TARDCS) involves high salt lysis of agarose-embedded unfixed cells, and thereby only preserves covalent DNA-drug and DNA-protein interactions [22]. DNase I and proteinase K treatments were incorporated into the assay to characterize the molecular targets of activated NIs. Addition of DNase I did not affect N_3_-AZA click staining, whereas cells treated with proteinase K lost all of their click signal (Fig. 4A and B). This suggests that almost all covalent binding of NIs under hypoxia was to proteins, and interaction between DNA and activated NIs, if any, was likely not covalent in nature.

To assess effects of NIs on DNA integrity, immunofluorescence staining for γ-H2AX was performed as a read-out of DNA damage (Fig. 4C). A significant increase in the percentage of cells with high γ-H2AX signal was seen in IAZA- and FAZA-treated hypoxic cells (Fig. 4D and E). Interestingly, when DNA damage was measured directly by single cell gel electrophoresis (alkaline comet assay), a dampened response was observed (Fig. 4F) Although a less pronounced (but significant) increase was seen with IAZA under hypoxia, FAZA treatment did not generate any difference when compared to vehicle-treated hypoxic cells. The poor association between γ-H2AX staining and alkaline comet data likely indicates a non-canonical phosphorylation of H2AX under hypoxia in response to NI treatment, which appears to be independent of DNA damage and can potentially be attributed to replication stress induced γ-H2AX formation.

Accumulation of ROS has previously been reported in response to NI treatment in glioma stem cells [28]. To check if IAZA/FAZA treatment can induce ROS generation, CVS assays were performed where cells were pre-incubated for 2 h with 3 mM NAC, a ROS scavenger, before drug treatment. The NAC concentration, chosen based on previous reports [38], was validated experimentally (Fig. S3F). Pre-treating cells with NAC did not improve viability in IAZA- and FAZA-treated hypoxic cells (Fig. S3A-**B**), which implies that these compounds do not generate toxic levels of ROS. Additionally, cellular ROS levels were also directly measured with a commercial kit. The kit uses a substrate that reacts with H_2_O_2_ to generate a luciferin precursor, which upon reaction with a detector reagent, is converted to luciferin and produces luminescence. Since the substrate must be added at the end of the hypoxic incubation, plates needed to be taken out of the hypoxia chamber, which inevitably led to reoxygenation. ROS generation was measured in cells that (after adding the H_2_O_2_ substrate) were either placed back in the hypoxia chamber, or were subjected to reoxygenation for the entire duration of substrate incubation (Fig. S3C). While drug-treated hypoxic cells did show a dose dependent increase in ROS levels when they were put back under hypoxia, the changes were not statistically significant (Fig. S3D-**E**). Also, due to the nature of the assay, the contribution of the brief reoxygenation on the ROS levels cannot be ruled out. A more potent and significant increase in ROS levels was observed in drug-treated hypoxic cells that were allowed to reoxygenate for 6 h (Fig. 4G and H). This implies that hypoxic incubation with IAZA and FAZA can promote a compromised state that facilitates enhanced ROS generation upon reoxygenation.

### 3.5 IAZA and FAZA treatment under hypoxia alter actin distribution and focal adhesion

**Fig. 5:**
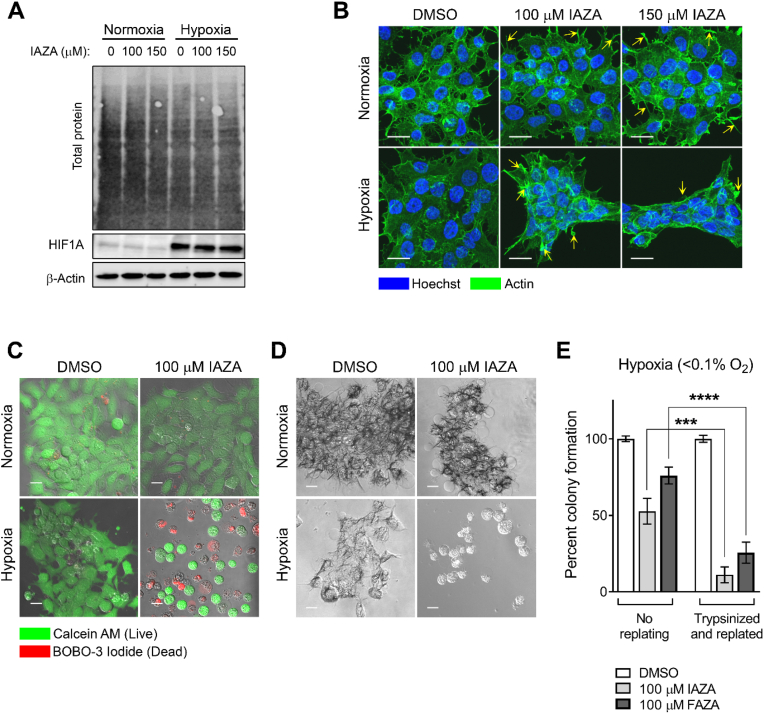
**Effects of IAZA treatment on actin and cell adhesion properties.** Extracts from FaDu cells treated with IAZA (100 μM or 150 μM) or vehicle control (0.02% DMSO) for 24 h under normoxia or hypoxia (<0.1% O_2_) were processed for β-actin immunoblotting; HIF1A was used to show successful induction of hypoxia. No significant difference was observed in total actin levels (**A**). Hypoxic cells treated with IAZA showed altered actin cytoskeleton with fewer and stunted cell projections (highlighted with yellow arrows) (**B**). Drug-treated hypoxic cells, upon replating, showed compromised adhesion (**C**), metabolic capacities (**D**) and colony forming capacities (**E**). Representative immunoblots and micrographs are shown; scale bar = 20 μm. Data show the mean from three independent experiments; error bars represent S.E.M. (For interpretation of the references to color in this figure legend, the reader is referred to the Web version of this article.)

Activated NIs covalently bind to cellular proteins in hypoxic cells. Using N_3_-AZA (a structural homolog of IAZA and FAZA), we have previously described a proteomic approach for identification of NI protein targets [20]. A total of 62 proteins were reported to form adducts with NIs, including actin, cortactin, tubulin, tropomyosin alpha-3 chain, transgelin-2 and desmoplakin-all of which are involved in cytoskeletal organization, adhesion and in providing structural integrity to cells. Considering the pronounced change in cell morphology in response to NI treatment (Fig. 2A), we sought to identify the role NI adduction plays on the cytoskeletal protein, actin. Drug treatment did not change actin protein levels (Fig. 5A), but overall, a reduction in cell projections as well as alterations in actin distribution was observed in drug-treated hypoxic cells (more obvious in the 150 μM group) (Fig. 5B). Actin staining in the periphery of IAZA-treated hypoxic cells appeared more prominent and condensed when compared to vehicle-treated hypoxic cells. To further investigate the effects on focal adhesion, cells were subjected to trypsinization and replated following treatment. Interestingly, drug-treated hypoxic cells undergoing proteolytic detachment showed reduced adhesion, even when they stain “live” based on intracellular esterase activity (Fig. 5C), and a complete loss of metabolic activity (assessed through their capacity to metabolize the tetrazolium dye, MTT) (Fig. 5D). Similar trends were obtained with FAZA (data not shown). Consequently, drug-treated hypoxic cells that underwent replating following treatment also showed a significant reduction in colony formation capacities when compared to cells that were not subjected to trypsinization (Fig. 5E).

### 3.6 Hypoxic incubation with IAZA and FAZA reduce targeted enzyme activities

**Fig. 6:**
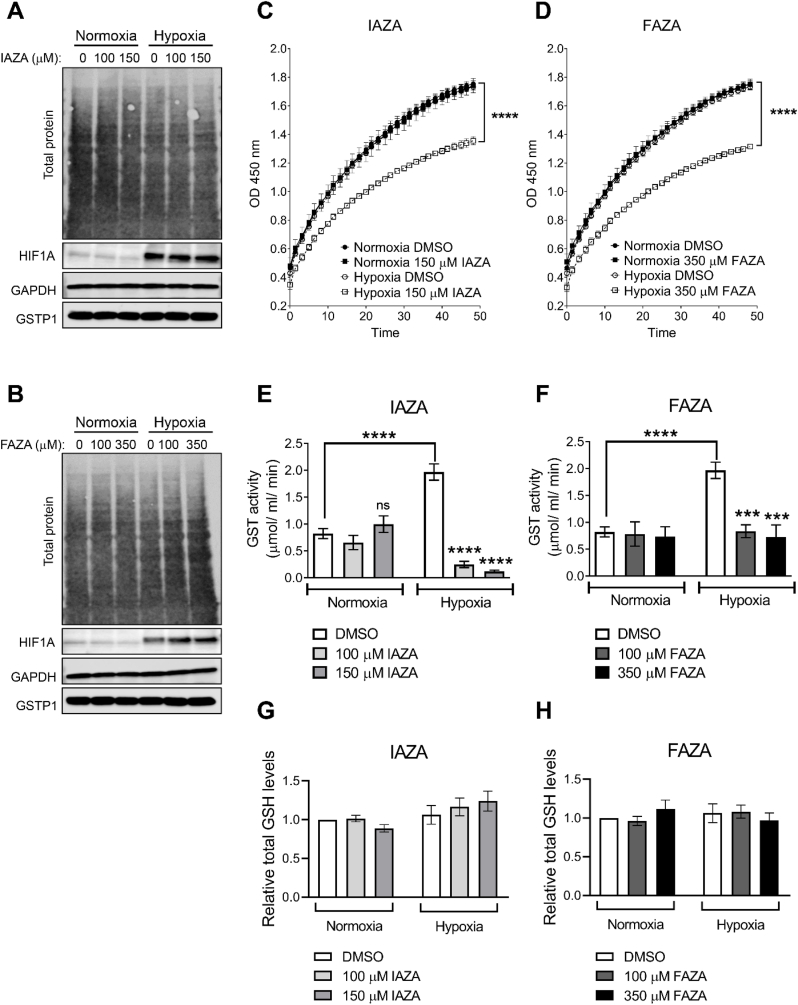
**The effects of IAZA and FAZA treatment on target proteins and glutathione levels under hypoxia.** FaDu cells treated with IAZA (**A**) and FAZA (**B**) were processed for GAPDH and GSTP1 immunoblotting showing no effects on the protein levels in response to drug treatment; HIF1A indicates successful induction of hypoxia. Drug treatment significantly reduced GAPDH (**C** and **D**) and GST (**E** and **F**) enzymatic activities only under hypoxia. The cellular GSH pool remained unchanged in response to IAZA (**G**) and FAZA (**H**) treatment. Representative Western blot images are shown. Mean ± S.E.M. from three independent experiments are shown.

In addition to the cytoskeletal proteins, key enzymes in the glycolytic (GAPDH) and cellular detoxification (GST) pathways are also targeted by NIs [20]. Previously, we have shown that N_3_-AZA disrupted the catalytic activities of GAPDH and GST without affecting total protein levels. Similar trends were observed in IAZA- and FAZA-treated cells, showing no effect on GAPDH and GST protein levels (Fig. 6A and B, S4). By assaying NADH levels in cell extracts (generated as a by-product of GAPDH-mediated oxidization of glyceraldehyde-3-phosphate to 1, 3-bisphosphoglycerate), we found that GAPDH activity was significantly reduced in IAZA- and FAZA-treated hypoxic cells (Fig. 6C and D). Total GST activity was measured using 1-chloro-2,4-dinitrobenzene (CDNB) that reacts with reduced glutathione and results in an increase in absorbance, which is directly proportional to the GST activity in the sample. IAZA and FAZA treatment dramatically reduced GST activity, but only under hypoxia. In comparison to the vehicle-treated hypoxic group, IAZA- and FAZA-treated hypoxic cells displayed ∼90% and ∼60% reduction in GST activity, respectively. GST appears to be more susceptible to IAZA/FAZA treatment than that of GAPDH, since a lower concentration of drug (100 μM) was enough to severely inhibit its catalytic activity (Fig. 6E and F). This phenomenon cannot be attributed to exhaustion in the cellular glutathione pool (another potential cellular target of NIs [6] and a substrate for GST) since total glutathione levels remained unaffected (Fig. 6G and H). To assess if inhibition of GAPDH and GSTP1 was directly responsible for the reduction in proliferation observed following treatment with IAZA and FAZA, we monitored the viability of cells in which GAPDH and GSTP1 were transiently depleted by siRNA. However, no effect on cell viability was observed using this approach (Fig. S5).

### 3.7 IAZA-treated mice showed an initial delay in tumour growth and a decrease in endpoint hypoxia levels

**Fig. 7:**
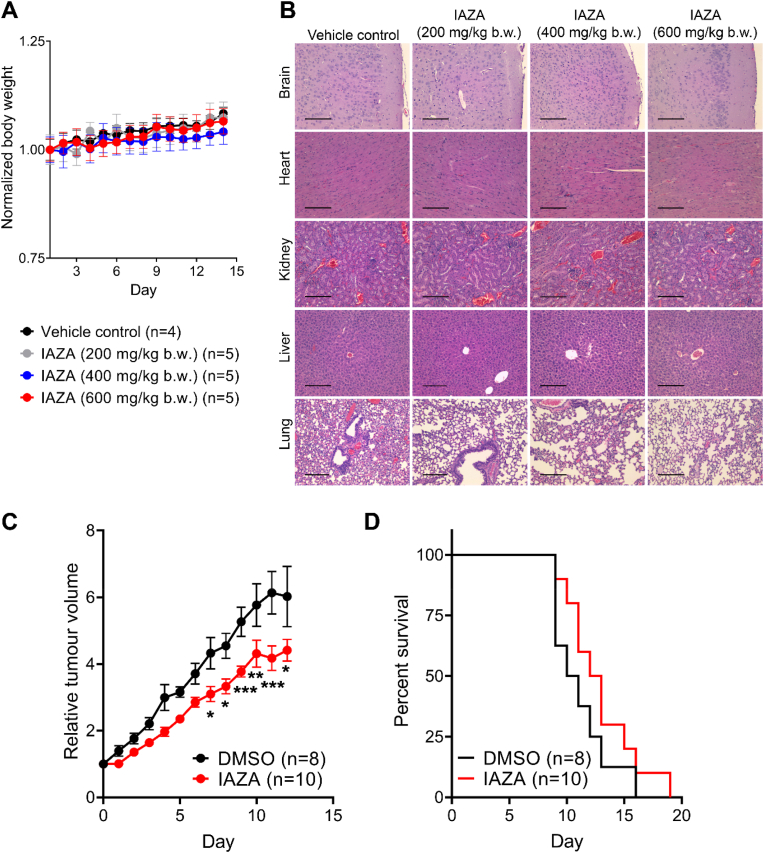
***In vivo* evaluation of IAZA toxicity and tumour growth delay properties.** IAZA was injected intraperitoneally in NOD/SCID/IL2R mice at 200, 400 or 600 mg/kg b.w. Changes in body weight were monitored for 14 days post injection (**A**), after which, mice were sacrificed, and histopathology analysis was performed on organ sections for possible signs of toxicity. Representative histopathology micrographs are shown (**B**). Tumour growth delay capacities of a single IAZA (400 mg/kg b.w.) administration was monitored in NU/NU nude mice bearing subcutaneous FaDu tumours. IAZA-treated mice showed an initial delay in tumour growth (**C**), with a small increase in duration of survival (**D**). Data show mean ± S.E.M.; scale bar = 1 mm (**B**).

Since IAZA showed a more robust response in *in vitro* experiments than FAZA, animal studies were carried out only with IAZA. To determine a safe dose for IAZA administration, a toxicity study in NOD/SCID/IL2R mice was performed with IAZA injected once intraperitoneally at 200 mg, 400 mg, or 600 mg per kg body weight. The drug was well tolerated, and no significant weight loss was observed in mice over 14 days post injection (Fig. 7A). Histopathological analysis showed no organ toxicity (Fig. 7B), and no significant differences were found in parameters analyzed for blood chemistry (Table S1).

After establishing a toxicity profile, a tumour growth delay experiment was performed where NU/NU nude mice bearing subcutaneous FaDu tumours (∼200–300 mm^3^) were injected with a single dose of 400 mg IAZA/kg b.w. Mice were monitored daily, tumour volume and body weight were recorded, and animals were sacrificed when the tumour volume reached ∼1500 mm^3^. Although IAZA-treated mice showed an initial delay in tumour growth (Fig. 7C), the effects diminished over time and did not result in any significant increase in survival (Fig. 7D). Body weight and tumour volume of individual mice are shown in Figs. S6A–D. To examine potential prolonged effects of IAZA on tumour hypoxia, tumour sections were processed for endpoint hypoxia levels using N_3_-AZA click chemistry, which was previously reported as a potent hypoxia marker comparable to pimodindazole immunostaining [20]. A statistically significant decrease in hypoxia levels was seen in tumours from the IAZA-treated group compared to the vehicle-treated group (Figs. S6E and F).

## 4 Discussion

Tumour hypoxia is widely acknowledged as a formidable barrier for effective radio- and chemotherapy and more recently immunotherapy. Hypoxic tumour cells can escape the toxic effects of standard treatment modalities, and thus play a direct role in disease recurrence [39,40]. Consequently, eradication of hypoxic tumour fractions can significantly potentiate treatment outcome. Regardless, hypoxia-directed therapeutic interventions have yet to become a part of routine clinical practice, mainly because few drugs are able to reach and remain in the diffusion limited regions within the tumour. Hypoxic cells are also quiescent in nature, which protects them from conventional anti-cancer drugs that usually target rapidly proliferating cells [41]. One promising strategy to subvert this challenge is to use hypoxia activated prodrugs (HAPs), which are compounds with select chemical moieties that allow them to preferentially accumulate and become activated in hypoxic tissue [42]. Nitroimidazoles (NIs), one of the most widely studied HAPs, garnered considerable attention as potential hypoxic anti-cancer drugs when the hypoxic radiosensitization properties of metronidazole (a 5-NI) was first reported in 1973 [43]. Despite showing encouraging results in pre-clinical models, several large-scale clinical trials with NIs failed to provide definitive therapeutic advantage. Nonetheless, there is merit to the idea that NIs can improve therapy outcome in a properly stratified patient population, as evident from the DAHANCA study with nimorazole [10,11]. Considering that NI compounds have been under investigation for decades now, it is surprising that their molecular mechanism is still poorly understood, which has constrained further development of NI-based anti-cancer therapeutics. Indeed, most NI research nowadays focuses on their utility as hypoxic radiotracers; only a handful of mechanistic studies with NIs have been reported in recent times, mostly in anaerobic microbes [[44], [45], [46]]. This warrants a methodological analysis of NIs in a human cancer model with a particular emphasis on elucidating the effects they have on hypoxic cancer cells.

Iodoazomycin arabinofuranoside (IAZA) and fluoroazomycin arabinofuranoside (FAZA) are 2-NI hypoxic radiotracers that have shown excellent tumour uptake in clinical studies, demonstrating their abilities to overcome the diffusion barrier to reach hypoxic tumour niches. In addition, these compounds have also shown therapeutic potential in cell based assays: FAZA has been reported as a hypoxic radiosensitizer in human colorectal cancer cells [18], while IAZA showed hypoxic cytotoxicity in murine breast cancer cells [50] and sensitized hypoxic human hepatocellular carcinoma cells to ionizing radiation (IR) [17]. The iodine moiety of IAZA makes it particularly promising for therapy; iodine in NIs has been reported to increase their hypoxic cytotoxicity [47]. It also makes IAZA a versatile molecule that can be labelled with an iodine radioisotope of choice for imaging (^123^I and ^124^I) and molecular radiotherapy (^131^I).

In this study, a human head and neck cancer (HNC) model was used to assess the therapeutic potentials of IAZA and FAZA because (a) HNC tumours harbor significant levels of hypoxia [48], (b) pre-treatment tumour oxygenation status in HNC patients dictates response to therapy [49], and (c) an improvement in HNC patient outcome was seen in a meta analysis of 50 randomized NI clinical trials [50]. First, the sensitivity of FaDu cells towards IAZA and FAZA treatment was tested under different O_2_ levels. In general, the lower the O_2_ levels, the higher the cells’ sensitivity was towards the drugs (Fig. 1D, E, G and **H)**. Depending on the assay, the IC_50_ values were ∼100–150 μM for IAZA and ∼300–350 μM for FAZA under ≤0.1% O_2_ levels. It is worth noting that cells in these O_2_ levels (otherwise known as radiobiological hypoxia) represent the most challenging population to target therapeutically [51]. Cells displayed higher sensitivity towards IAZA than FAZA, most likely due to the higher lipophilicity of IAZA, which might aid in its enhanced cellular retention [13]. This trend remained consistent across a panel of different human cancer cell lines (Fig. S1). Since NIs, at high concentrations, are known to induce neurotoxicity [52], a conscious decision was made to limit the test concentrations in the micromolar range and close to 100 μM when possible.

Interestingly, while morphological changes with IAZA and FAZA treatment were clearly evident under hypoxia (Fig. 2A), no significant increase in apoptotic (Fig. 2B and C), necrotic (Fig. 2D) or senescent (Fig. 2F) populations was recorded. Unlike doranidazole (another 2-NI) [28], IAZA and FAZA treatment (at the concentrations tested) did not induce ferroptosis under hypoxia (Fig. 2G and H). Instead, hypoxic treatment with 100 μM IAZA or FAZA generated an inhibitory effect on cell proliferation (Fig. 2I and J). Cell cycle analysis showed a significant increase in S phase population in drug-treated hypoxic cells (Figs. 3A and S2A), which was further corroborated by staining for chromatin bound PCNA (Fig. 3B and C) and cyclin E1 protein levels (Fig. S2B and **C)**. An increase in S phase population arose not because more cells were actively dividing, rather hypoxic cells treated with the drugs appeared to progress through the S phase at a slower rate than the vehicle-treated cells. DNA replication was severely compromised in IAZA- and FAZA-treated hypoxic cells, which was analyzed by monitoring the uptake of a nucleoside analogue, EdU (Fig. 3D and E). The inhibitory effects of IAZA and FAZA on DNA synthesis are in agreement with previous reports using NIs in anaerobic bacteria [53,54]. Delay in DNA replication in drug-treated hypoxic cells induced replication stress, observed through nascent fork degradation in the DNA fibre assay (Fig. 3G), and further confirmed by analyzing retention of chromatin bound RPA in the nucleus. While the intensity of RPA staining in IAZA-treated hypoxic cells was almost double (and statistically significant) than the vehicle-treated hypoxic cells, FAZA treatment (at 100 μM) resulted in a non-significant increase in RPA staining (Figs. S2E and F). Importantly, withdrawal of drugs and subsequent reoxygenation did not immediately reverse the phenotype; drug-treated hypoxic cells maintained a slower rate of DNA replication (Figs. 3H and S2D), cell cycle progression (Fig. 3A) and proliferation (Fig. 2I and J).

In relation to the effects seen with IAZA and FAZA treatment on DNA synthesis under hypoxia, it is worth noting that these compounds are structural analogs of nucleosides, and previously FAZA has been reported to bind to human concentrative nucleoside transporters 1–3 (hCNT1-3) [55]. While our data suggest that such interactions can compete with or block nucleoside transport, evident from a reduction in EdU uptake in response to treatment (Fig. 3D and E), the specificities of this process are not completely understood. For example, it is not clear if the drugs need to be bioreduced to bind to hCNTs. Maier et al. reported binding under normal O_2_ conditions (employing *Saccharomyces cerevisiae* with recombinant hCNTs as their model) [55], but in FaDu cells, the effects did appear to be hypoxia selective, which suggests that bioreductive activation might play a role in the process. Binding of IAZA and FAZA to nucleoside transporters and subsequent deregulation in the transporters’ activities may provide a potential explanation for the compromised DNA synthesis and slower proliferation rates observed in drug-treated hypoxic cells. However, this phenomenon will likely be unique to only sugar coupled NIs, such as IAZA and FAZA, and not a general mechanism for all NIs.

Formation of DNA-NI adducts [56] and/or subsequent DNA damage [35] has often been proposed as contributing factors for enhanced sensitivity of hypoxic cells towards NI treatment. Although direct binding of activated NIs to DNA has been reported earlier [57], the exact nature and to what degree this binding happens require further investigation. To assess DNA-NIs binding in hypoxic cells, a modified TARDIS assay (called TARDCS) was employed that utilizes N_3_-AZA click chemistry. The TARDCS assay only captures covalent interactions of drug or macromolecules to DNA, and N_3_-AZA allows for fluorescent labelling of NI-adducts formed under hypoxia through click chemistry. Analysis of N_3_-AZA treated normoxic and hypoxic cells showed that covalent N_3_-AZA-adducts were only formed under hypoxia (Fig. 4A). Addition of DNase I had a minimal effect on the N_3_-AZA click signal, while proteinase K treatment completely removed all click staining (Fig. 4A and B). This implies that proteins, rather than DNA, are primary NI targets for covalent adduct formation. Since removal of proteins by proteinase K treatment removed all click staining, any DNA-NI interaction, if it occurs, would have to be non-covalent. While our observations are at odds with findings by Varghese et al. [58], recent evidence suggests that NI binding to DNA may potentially occur through hydrogen bonding and electrostatic interaction, instead of covalent association [59].

The capacity of activated NIs to directly induce DNA damage has been contested lately, with NI-induced DNA damages in earlier *in vitro* reports being attributed to the use of very high concentrations of drugs that are not achievable *in vivo* (reviewed in Ref. [60]). The effects of IAZA and FAZA treatment on DNA integrity in the present study were assessed using γ-H2AX immunofluorescent staining (Fig. 4C) and alkaline comet assay (Fig. 4F). While drug-treated hypoxic cells showed an increase in the percentage of γ-H2AX positive population (Fig. 4D and E), a relatively lower level of DNA damage was seen in the comet assay (Fig. 4F). It should be noted here that replication stress can also induce phosphorylation of H2AX in the absence of DNA damage [61]. Given the concentrations used in our assays and that IAZA and FAZA induce replication stress under hypoxia (Fig. 3G, **S2E** and **F**), the high γ-H2AX staining in this population is most likely to be a consequence of replication stress rather than DNA damage. Additionally, we also analyzed generation of ROS in response to IAZA/FAZA treatment using two different approaches. Pre-incubating cells with a ROS scavenger (NAC) did not alter cell viability in response to IAZA/FAZA treatment under hypoxia, suggesting that they do not elevate ROS levels (Figs. S3A and B). However, IAZA- and FAZA-treated hypoxic cells, when reoxygenated, showed significantly high levels of H_2_O_2_, indicating that 2-NI treatment generated a compromised state in hypoxic cells that makes them susceptible to reoxygenation mediated stress (Fig. 4G and H). Considering that tumours often display dynamic oxygenation with fluctuating O_2_ concentrations (termed cyclic hypoxia) (reviewed in Ref. [62]), elevated ROS levels in reoxygenated cells treated with NIs might have significant biological implications.

Our data indicate that proteins are likely the primary targets of activated NIs for covalent adduct formation under hypoxia. To date, only a few studies have explored the proteins that NIs bind to. Consequently, the effects these interactions have on target protein stability, turnover, or function are poorly understood. Previously, we described a proteomic approach with N_3_-AZA to identify NI target proteins, and reported a total of 62 candidate targets with important biological functions [20]. Given the distinct change in cell morphology in response to IAZA and FAZA treatment under hypoxia, we chose to study the cytoskeletal protein actin (which is a target of NIs [20]) in respect to total protein levels and microfilament organization. While total actin levels remained unchanged (Fig. 5A), the number of cellular projections appeared to be reduced in drug-treated hypoxic cells, with condensed staining of the actin filaments in the cell periphery (Fig. 5B). Although we did not see complete depolymerization of actin fibers, hypoxic cells treated with drugs struggled to establish functional anchorage to the extracellular matrix (ECM) when subjected to trypsinization (Fig. 5C). Focal adhesion of cells to the ECM primarily occurs through interactions between actin filament bundles and ECM protein fibronectin, and is mediated by α-actinin, vinculin, talin and integrin (a receptor for fibronectin) [63]. Interestingly, Cys374 residue on actin (the only one exposed out of the 6 conserved Cys residues in actin molecule) plays a crucial role in this process; it serves as a binding site for protein disulfide-isomerase (PDI, also a target for NIs [20]), which is essential for cell adhesion to fibronectin via integrin [64]. Interestingly, Cys has often been proposed as the site of adduct formation for NIs [65], and therefore, further investigation is warranted to determine if Cys374 on actin is modified by IAZA and if this would affect actin interaction with fibronectin.

In addition to the cytoskeletal proteins, many other proteins involved in critical biological functions related to hypoxic stress (such as the glycolytic enzyme GAPDH and the detoxification enzyme GST) form adducts with NIs [20]. In agreement with our results with N_3_-AZA treatment, GAPDH and GST protein levels remained unaffected by IAZA and FAZA treatment, regardless of O_2_ levels (Fig. 6A and B, S4). Covalent attachment of the NIs appears to primarily affect the enzymatic function of these target proteins; both GAPDH and GST proteins significantly lost their catalytic activities in response to IAZA and FAZA treatment under hypoxia (Fig. 6C–F). GAPDH and GST play important roles in hypoxia adaptation, and there are direct consequences of their inhibition on cell viability. For example, GAPDH depletion affects proliferation and induces cell cycle arrest in human lung, renal and colorectal cancer cells [66,67]. On the other hand, hypoxic cells elevate GST activity to counteract the effects of altered intracellular redox state [68], and siRNA-mediated knockdown of GSTP in HNC cell lines, HSC3 and SAS, suppressed cell growth by 50.5% and 35.2%, respectively [69]. To determine if inhibition of GAPDH and GST functions by IAZA and FAZA under hypoxia directly contributes to reduced cell proliferation, we transiently knocked down both proteins (either individually or together) (Fig. S5A), but this did not result in reduced cell growth (Fig. S5B), suggesting that suppression of GAPDH and GST catalytic activities by IAZA and FAZA is likely not sufficient to inhibit FaDu cell proliferation. However, it must be borne in mind that siRNA-mediated knockdown of protein levels does not necessarily mimic pharmacological inhibition [70]. In addition, it should be noted that our proteomic analysis identified 60 other protein targets of N_3_-AZA [20], and their contribution to the observed hypoxic cellular sensitivity has yet to be fully explored. For example, our analysis identified chaperone proteins such as Hsp60, Hsp70 and Hsp90 as NI targets, which are known to be involved in hypoxia-responsive cellular proliferation [71]. Finally, we cannot rule out the possibility that the hypoxia selective sensitization phenomenon by IAZA and FAZA may very likely be a combined effect of targeting many different pathways and cellular processes, and not a distinct isolated occurrence.

IAZA, administered intraperitoneally, was well tolerated in mice at levels up to 600 mg/kg b.w. (Fig. 7A and B, Table S1). Although a single dose administration of IAZA (400 mg/kg b.w.) to tumour bearing mice had minimal effect on overall survival (Fig. 7D), a significant delay in initial tumour growth was observed (Fig. 7C). While these early effects gradually wore off, our results are still promising when taken into consideration that only a single dose of IAZA was administered to mice, while most anti-cancer drugs (including nimorazole) are given as multiple doses in a clinical setting. Indeed, multiple administrations of IAZA may prove useful at improving survival, which needs to be tested experimentally in future. Additionally, administration of encapsulated IAZA might be another approach to ensure maximum efficacy of IAZA delivery to target tumour sites. Nanoencapsulation of IAZA and its benefits have been characterized in a previous study [17].

A major limitation of our animal data is that the pre-treatment tumour oxygenation status was not known. This makes it difficult to conclusively attribute the therapeutic gains directly to IAZA. Nonetheless, even in this unstratified population, IAZA administration had a long-term effect and resulted in a significant decrease in endpoint hypoxia levels (Figs. S6E and F), which may have clinical implications. However, how IAZA-treated tumours became less hypoxic requires further exploration.

In conclusion, we show that activated 2-NIs, IAZA and FAZA, alter cell morphology, negatively affect DNA synthesis and cell cycle progression under hypoxia, leading to slower proliferation of hypoxic NI-treated cells. Covalent binding of these NIs to proteins can inhibit catalytic functions of critical cellular enzymes such as GAPDH and GST. However, it was not possible to directly attribute the drug-induced cytostasis to the reduced activity of these two enzymes. Further analysis of other proteins targeted by IAZA and FAZA will be required to fully define the mechanisms responsible for altered cell morphology and proliferation. IAZA, even at a single i.p. injection, slowed initial tumour growth with no adverse effects.
