# Supplementary material for: Cellular mechanism of action of 2-nitroimidazoles as hypoxia-selective therapeutic agents
Source: Redox Biol. 2022 Mar 21;52:102300. doi: 10.1016/j.redox.2022.102300 (PMC9038562; doi:10.1016/j.redox.2022.102300)
Supplement: Multimedia component 1 [file mmc1.pdf]

Supplemental Information

Cellular mechanism of action of 2-nitroimidzoles as hypoxia-selective therapeutic agents

Faisal Bin Rashed[a], Diana Diaz-Dussan[b], Fatemeh Mashayekhi[a], Dawn Macdonald[a], Patrick Nicholas Nation[c], Xiao-Hong Yang[a], Sargun Sokhi[a], Alexandru Cezar Stoica[a], Hassan El-Saidi[a,d], Carolynne Ricardo[a], Ravin Narain[b], Ismail Hassan Ismail[a,e], Leonard Irving Wiebe[a], *Piyush Kumar[a] and *Michael Weinfeld[a]

[a] Department of Oncology, University of Alberta, Edmonton, Alberta, AB T6G 2R3, Canada

[b] Department of Chemical & Materials Engineering, University of Alberta, Edmonton, Alberta, AB T6G 2R3, Canada

[c] Department of Laboratory Medicine & Pathology, University of Alberta, Edmonton, Alberta, AB T6G 2R3, Canada

[d] Department of Pharmaceutical Chemistry, Faculty of Pharmacy, University of Alexandria, El Sultan Hussein St. Azarita, Alexandria, Egypt

[e] Biophysics Department, Faculty of Science, Cairo University, Cairo, Egypt

***Correspondence:**

Michael Weinfeld
Department of Oncology
University of Alberta
116 St & 85 Ave, Edmonton, AB T6G 2R3, Canada
E-mail: mweinfel@ualberta.ca

Piyush Kumar
Department of Oncology
University of Alberta
116 St & 85 Ave, Edmonton, AB T6G 2R3, Canada
E-mail: pkumar@ualberta.ca

Table S1. Blood chemistry analysis of mice injected i.p. with IAZA (or vehicle control) showed no significant changes. Data represent mean± standard deviation.

	**Vehicle control**	**IAZA (200 mg/kg)**	**IAZA (400 mg/kg)**	**IAZA (600 mg/kg)**
Glucose (mmol/L)	17.65±4.52	18.22±3.98	17.14±3.20	16.64±1.76
Urea (mmol/L)	8.975±0.64	7.72±0.56	7.38±1.19	7.06±0.48
Creatinine (µmol/L)	9±3.37	9.2±1.30	8.4±2.19	9±1.41
Phosphorus (mmol/L)	3.85±0.42	3.82±0.16	3.68±0.53	3.76±0.34
Calcium (mmol/L)	2.51±0.24	2.594±0.18	2.574±0.07	2.588±0.02
Sodium (mmol/L)	154.5±1.73	152.6±2.19	156.5±2.65	154.6±3.05
Potassium (mmol/L)	>9	>9	7.2 to >9	8.8 to >9
Chloride (mmol/L)	114.5±3.70	114.8±3.11	114±2.12	114.6±0.89
Bicarbonate (mmol/L)	22.75±1.71	21.8±1.30	23.8±2.17	21.2±3.42
Total Protein (g/L)	41.5±9.33	44.4±6.43	47.2±2.59	46.2±1.92
Albumin (g/L)	24.25±5.0	26.4±3.58	27.8±1.10	27.4±0.89
Globulin (g/L)	17.25±4.35	18±2.92	19.4±1.52	18.8±1.30
ALT[a] (IU/L)	78.5±82.33	48.6±36.32	114.8±206.98	26.8±13.94
AST[b] (IU/L)	373.5±334.34	219.2±211.69	230.4±375.32	107.4±112.30
ALP[c] (IU/L)	95.25±26.16	98.8±12.44	61.8±7.53	81±5.70
Total Bilirubin (µmol/L)	3.2±0.91	3.98±0.65	4.46±0.64	4.8±0.75
CK[d] (IU/L)	13505±15265.23	8194±10978.56	342±427.05	279.25±238.97

[a] ALT: alanine aminotransferase; [b] AST: aspartate aminotransferase; [c] ALP: alkaline phosphatase; [d] CK: Creatine Kinase

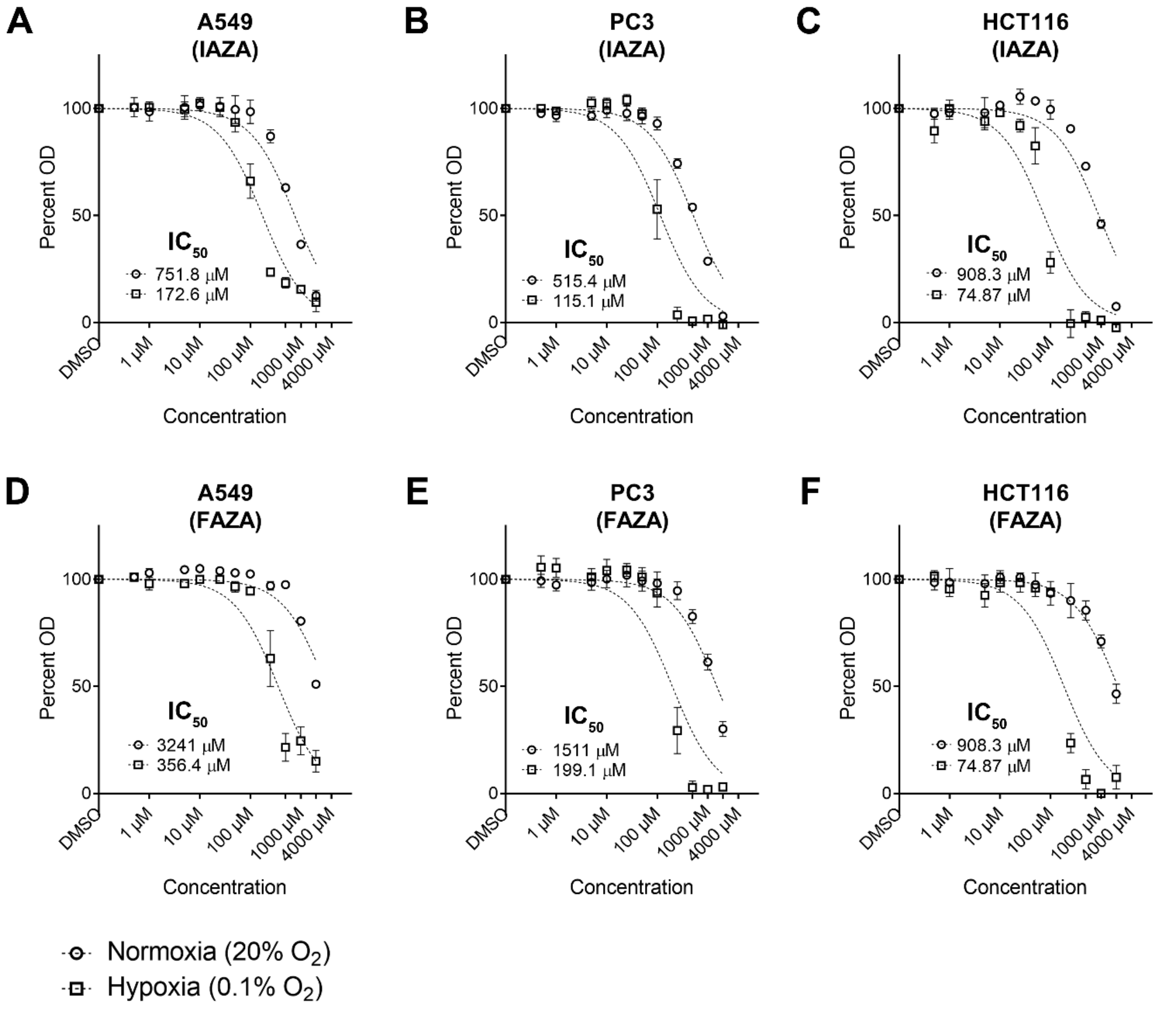

Figure S1. Hypoxia selective sensitivity to IAZA and FAZA in additional human cancer cell lines. Crystal violet staining assays were performed to assess sensitivity of human lung epithelial carcinoma A549 (**A, D**), prostate carcinoma PC3 (**B, E**) and colorectal carcinoma HCT116 (**C, F**) cells to IAZA (**A-C**) and FAZA (**D-F**) treatment under normoxia and hypoxia (0.1% O_2). Hypoxic cells showed higher sensitivity to drug treatment. Data show mean ± S.E.M. from three independent experiments.

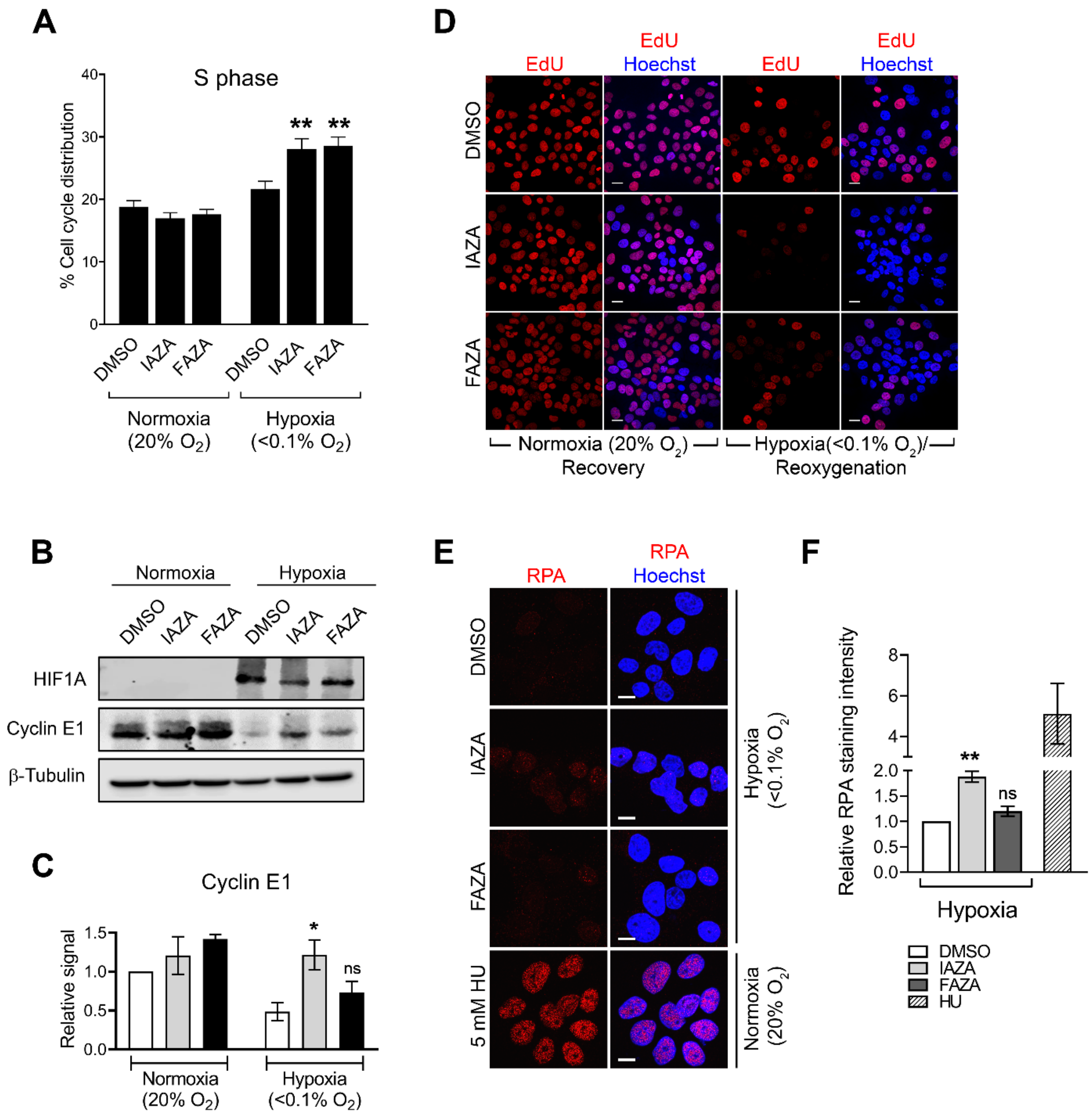

A
S phase
% Cell cycle distribution
0
10
20
30
40
**
**
DMSO
IAZA
FAZA
DMSO
IAZA
FAZA
Normoxia (20% O2)
Hypoxia (<0.1% O2)
D
EdU
EdU Hoechst
EdU
EdU Hoechst
DMSO
IAZA
FAZA
Normoxia (20% O2) Recovery
Hypoxia(<0.1% O2)/ Reoxygenation
B
Normoxia
Hypoxia
DMSO
IAZA
FAZA
DMSO
IAZA
FAZA
HIF1A
Cyclin E1
β-Tubulin
C
Cyclin E1
Relative signal
0.0
0.5
1.0
1.5
*
ns
Normoxia (20% O2)
Hypoxia (<0.1% O2)
E
RPA
RPA Hoechst
DMSO
IAZA
FAZA
5 mM HU
Hypoxia (<0.1% O2)
Normoxia (20% O2)
F
Relative RPA staining intensity
0.0
0.5
1.0
1.5
2.0
4
6
8
**
ns
Hypoxia
DMSO
IAZA
FAZA
HU

Figure S2. IAZA- and FAZA-treated hypoxic cells have higher S phase population, higher cyclin E1 levels, compromised DNA replication capacity even after reoxygenation, and increased chromatin-bound RPA. Hypoxic incubation with IAZA and FAZA induced a statistically significant increase in S phase population (**A**). Hypoxic cells treated with IAZA and FAZA had higher levels of cyclin E1, when compared to vehicle-treated hypoxic cells (**B** and **C**); a stronger signal in normoxic samples is probably reflective of more cells in late G1 phase (see **Fig. 3A**). Incorporation of EdU during reoxygenation and recovery was compromised in drug-treated cells (**D**). FaDu cells treated with IAZA (100 µM), FAZA (100 µM), hydroxyurea (5 mM) or vehicle control (0.02% DMSO) for 24 h under normoxia or hypoxia (<0.1% O_2) were processed for immunocytochemistry by probing for replication protein A2 (RPA). Salt-detergent extraction of cells prior to fixation ensures that only genomic DNA and any tightly bound proteins remain, while other cellular constituents are removed. The resultant RPA staining represents chromatin-bound RPA fraction (**E**). Quantification of nuclear RPA staining is shown in graph (**F**). Representative micrographs are shown; scale bar = 20 µm (**C** and **D)**; data represent mean ± S.E.M. from three independent experiments.

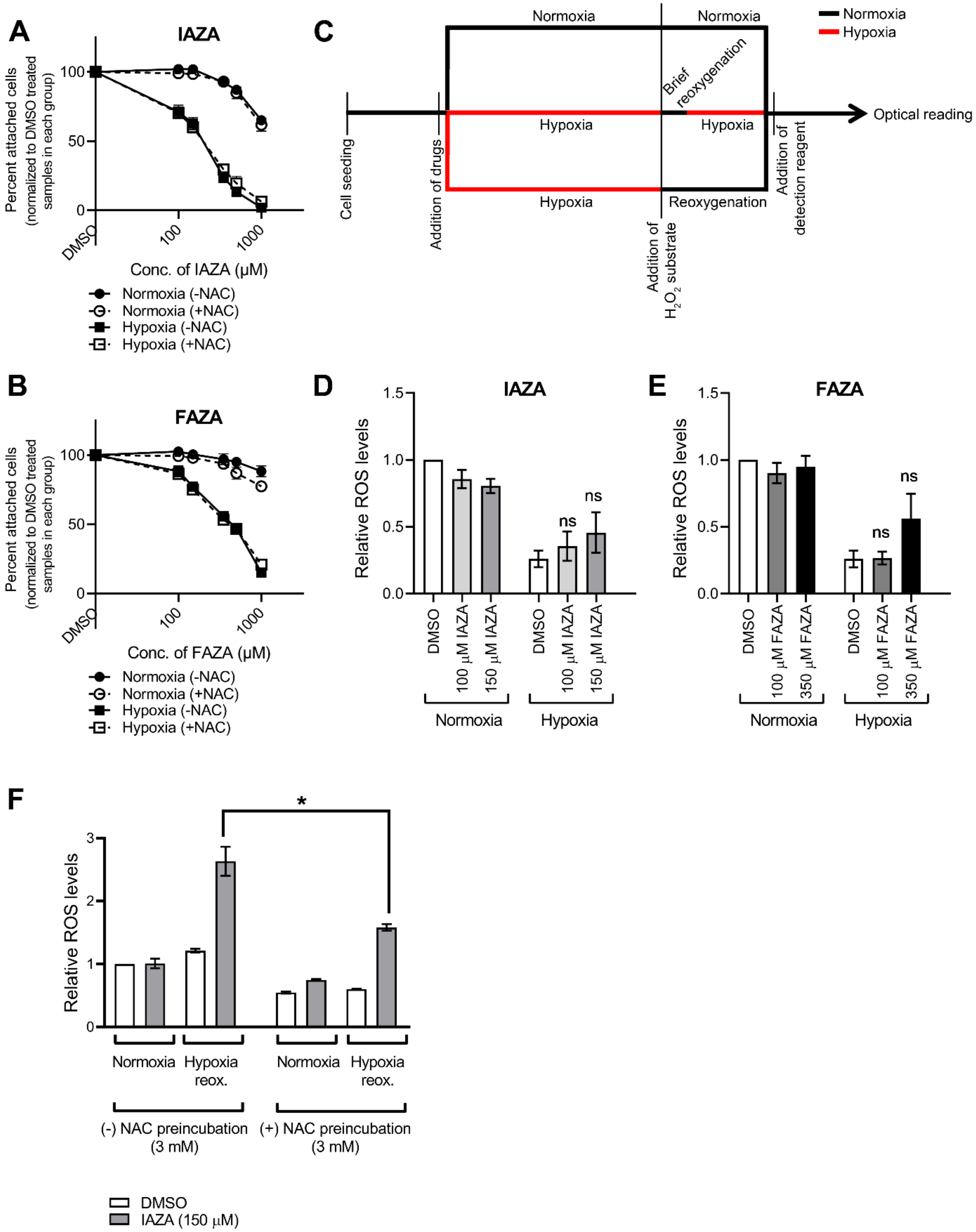

A
IAZA
Percent attached cells (normalized to DMSO treated samples in each group)
100
50
0
DMSO
100
1000
Conc. of IAZA (µM)
Normoxia (-NAC)
Normoxia (+NAC)
Hypoxia (-NAC)
Hypoxia (+NAC)
B
FAZA
Conc. of FAZA (µM)
C
Normoxia
Hypoxia
Brief reoxygenation
Reoxygenation
Optical reading
Cell seeding
Addition of drugs
Addition of H2O2 substrate
Addition of detection reagent
D
IAZA
Relative ROS levels
1.5
1.0
0.5
0.0
DMSO
100 µM IAZA
150 µM IAZA
ns
Normoxia
Hypoxia
E
FAZA
100 µM FAZA
350 µM FAZA
F
*
3
2
1
0
Hypoxia reox.
(-) NAC preincubation (3 mM)
(+) NAC preincubation (3 mM)
DMSO
IAZA (150 µM)

Figure S3. Analysis of ROS generation in response to IAZA and FAZA treatment under hypoxia. FaDu cells, preincubated with 3 mM NAC, were processed for the CVS staining assay with IAZA (**A**) and FAZA (**B**); cell viability did not change in response to NAC treatment. Experimental design for directly measuring cellular ROS levels (**C**); a full description of the experimental setup is provided in "Materials and methods" section. After addition of H_2O_2 substrate, if hypoxic drug-treated cells were placed back under hypoxia, a dose dependent non-significant increase in ROS levels were observed (**D** and **E**). Pre-incubation with 3 mM NAC decreased reoxygenation mediated ROS levels in IAZA-treated cells (**F**). Data show mean from three independent experiments; error bars represent S.E.M.

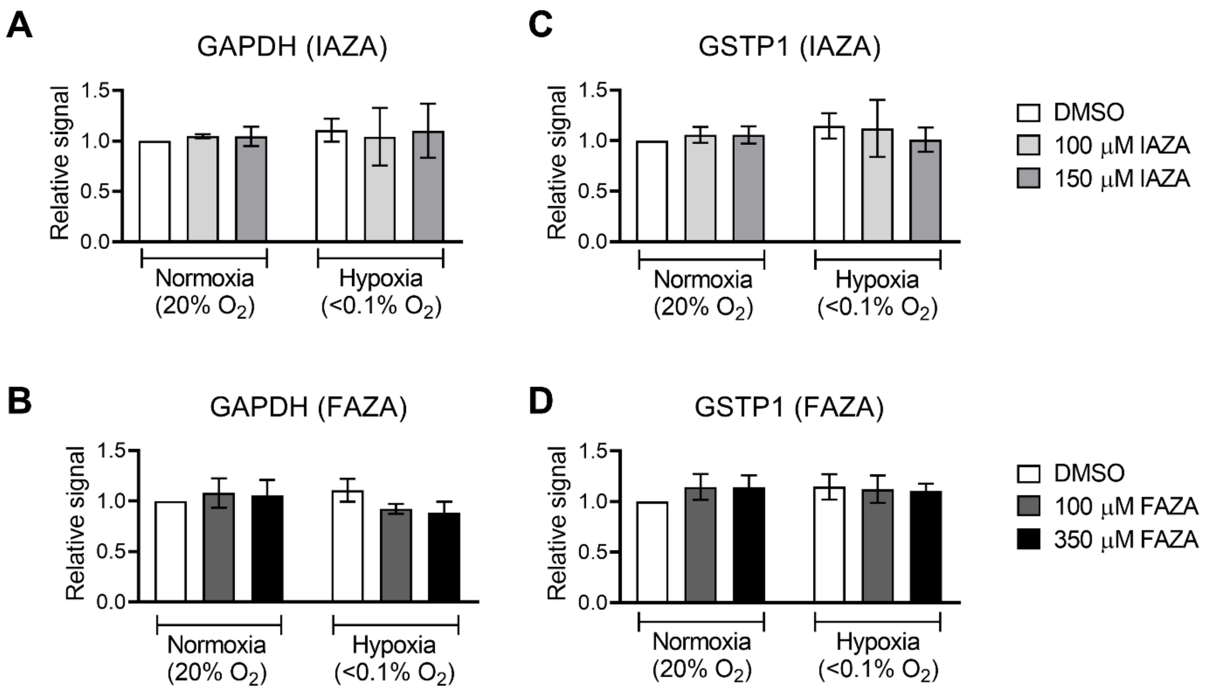

Figure S4. Quantification of GAPDH and GST protein levels in IAZA- and FAZA-treated cells. Extracts of FaDu cells treated with IAZA, FAZA or vehicle control (DMSO) were immunoblotted for GAPDH and GSTP1. Band intensities were measured using Image Studio lite and normalized to the vehicle-treated normoxic lane. No statistically significant differences were seen in GAPDH (**A** and **B**) and GSTP1 (**C** and **D**) protein levels in response to treatment. Data show mean from three independent experiments; error bars represent S.E.M.

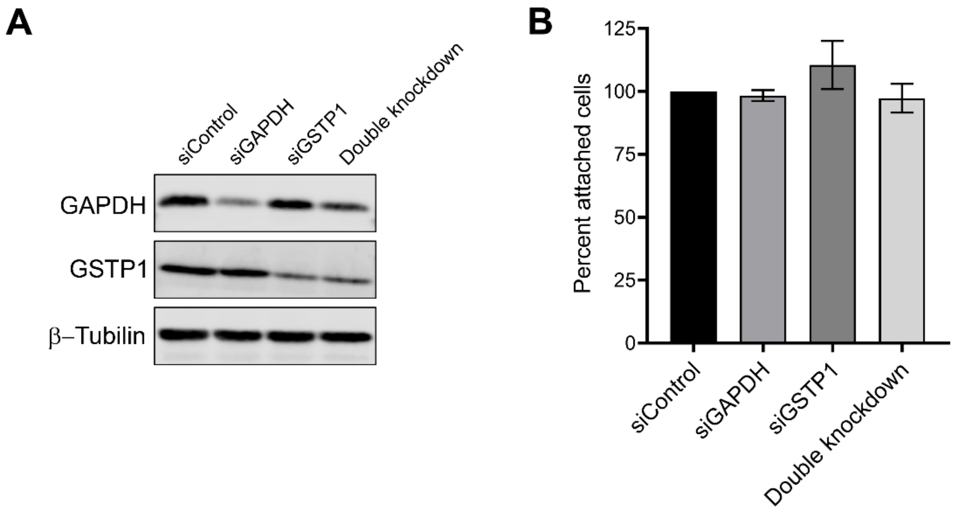

Figure S5. Effects of knock down of GAPDH and GSTP1 on cell growth. Extracts from FaDu cells transfected with siRNAs targeting GAPDH and GSTP1, either individually or in combination, were processed for immunoblotting. Control cells were transfected with a pool of non-targeting siRNAs. Knockdown efficiency was ~55% for GAPDH and ~62% for GSTP1. Representative immunoblots are shown from three independent experiments. (**A**). Viability of FaDu cells transfected as mentioned above was assessed by crystal violet staining. No significant difference was observed in cell viability among different groups (**B**). Data show mean from three independent experiments; error bars represent S.E.M.

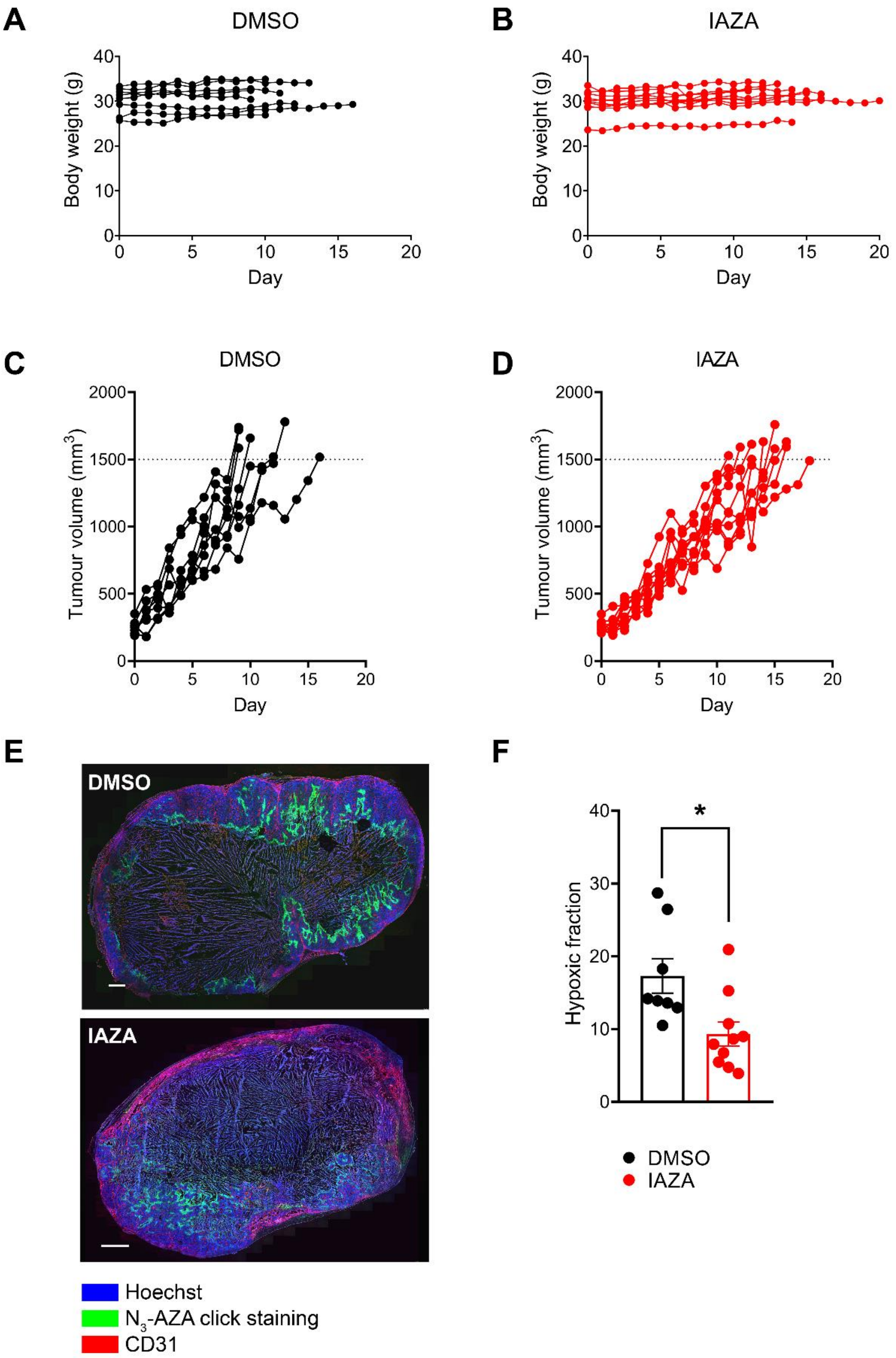
A
DMSO
Body weight (g)
Day
B
IAZA
Body weight (g)
Day
C
DMSO
Tumour volume (mm^3)
Day
D
IAZA
Tumour volume (mm^3)
Day
E
DMSO
IAZA
Hoechst
N_3-AZA click staining
CD31
F
*
Hypoxic fraction
DMSO
IAZA

Figure S6. Monitoring individual mice for body weight, tumour growth and endpoint hypoxia in response to IAZA treatment. NU/NU nude mice bearing subcutaneous FaDu tumours were injected i.p. with 400 mg/kg b.w. IAZA (or vehicle control, DMSO) once the tumours reached ~200-300 mm^3. Body weight (**A** and **B**) and tumour volumes (**C** and **D**) were monitored daily, until the tumours reached a volume of ~1500 mm^3, when mice were euthanized. Endpoint hypoxia levels were analyzed using N_3-AZA click staining; representative tumour sections are shown (**E**). Quantification of hypoxic fraction showed a significant reduction in hypoxia levels in tumours from IAZA-treated mice when compared to tumours from vehicle-treated mice (**F**). Data show mean± S.E.M. (**F**); scale bar = 1 mm (**E**).
